# Cold tolerance is unaffected by oxygen availability despite changes in anaerobic metabolism

**DOI:** 10.1038/srep32856

**Published:** 2016-09-13

**Authors:** Leigh Boardman, Jesper G. Sørensen, Vladimír Koštál, Petr Šimek, John S. Terblanche

**Affiliations:** 1 Department of Conservation Ecology and Entomology, Centre for Invasion Biology, Stellenbosch University, Private Bag X1, Matieland, 7602, South Africa; 2 Section for Genetics, Ecology & Evolution, Department of Bioscience, Aarhus University, Ny Munkegade 116, DK-8000 Aarhus C, Denmark; 3 Institute of Entomology, Biology Centre of the Czech Academy of Sciences, České Budějovice, Czech Republic

## Abstract

Insect cold tolerance depends on their ability to withstand or repair perturbations in cellular homeostasis caused by low temperature stress. Decreased oxygen availability (hypoxia) can interact with low temperature tolerance, often improving insect survival. One mechanism proposed for such responses is that whole-animal cold tolerance is set by a transition to anaerobic metabolism. Here, we provide a test of this hypothesis in an insect model system (*Thaumatotibia leucotreta*) by experimental manipulation of oxygen availability while measuring metabolic rate, critical thermal minimum (CT_min_), supercooling point and changes in 43 metabolites in moth larvae at three key timepoints (before, during and after chill coma). Furthermore, we determined the critical oxygen partial pressure below which metabolic rate was suppressed (*c*. 4.5 kPa). Results showed that altering oxygen availability did not affect (non-lethal) CT_min_ nor (lethal) supercooling point. Metabolomic profiling revealed the upregulation of anaerobic metabolites and alterations in concentrations of citric acid cycle intermediates during and after chill coma exposure. Hypoxia exacerbated the anaerobic metabolite responses induced by low temperatures. These results suggest that cold tolerance of *T. leucotreta* larvae is not set by oxygen limitation, and that anaerobic metabolism in these larvae may contribute to their ability to survive in necrotic fruit.

Common environmental stressors faced by insects include variation in oxygen availability, temperature and moisture^1^,^2^. For example, insects living at high altitudes or under ice will experience low temperatures and hypoxic (low oxygen) conditions^3^,^4^, while those living underground or in dung pats may experience hypoxic and/or hypercapnic (high carbon dioxide) conditions^5^. Similarly, some life-stages of insects (e.g. holometabolous larvae) may routinely experience hypoxic or anoxic conditions during development^6^. For most ectotherms, including many insects, fitness is reduced below optimal temperatures^2^,^7^. As temperatures decrease, voluntary movement typically becomes suppressed one to several degrees before reaching their critical thermal minimum (CT_min_), defined as the loss of co-ordinated movement^8^,^9^. The CT_min_ is the lowest limit of activity and therefore represents a functional, though not necessarily lethal, limit. At temperatures below CT_min_, insects enter an inactive coma like state, characterised by the absence of neurological activity (see e.g.^10^; review of mechanisms^9^; review of terminology and behavioural states^8^).

The proposed mechanisms underlying insect chill coma can be grouped into three main categories: i) whole-organism oxygen limitation, ii) signal transmission failure and iii) disruption of ion regulation^9^. Whole-organism oxygen limitation is based upon the hypothesis of oxygen- and capacity-limited thermal tolerance (OCLTT)^11^, which posits that oxygen limitation is the primary factor determining thermal tolerance (i.e. imposing a system-level constraint). According to the OCLTT hypothesis, once aerobic capacity has been exhausted at temperatures approaching the critical thermal limits, anaerobic mitochondrial metabolism begins and anaerobic by-products accumulate. This hypothesis was developed on data from marine animals, and its broader applicability to insects and other arthropods remains contentious and in urgent need of further research^12^,^13^,^14^,^15^,^16^. Aside from the proposed direct mechanisms of OCLTT, low oxygen availability may cause indirect stress as insects likely keep their spiracles open for longer to meet their constant cellular oxygen demands^17^, which in turn may result in elevated respiratory water loss rates^18^,^19^,^20^. Increasing metabolic rate - or sustained opening of the spiracles at a given ambient oxygen concentration - may also result in oxidative damage, assuming that cellular respiration rates remain constant. These indirect changes can in turn affect CT_min_ and low temperature tolerance by influencing osmotic balance and, consequently, ion homeostasis and nerve transmission^10^. In addition, anoxia may affect the plasticity of cold tolerance in various Diptera species. Rapid cold hardening (RCH) is a form of phenotypic plasticity whereby a non-lethal cold “shock” increases the insects’ chill tolerance^21^. While anoxia was able to elicit RCH in the house fly *Musca domestica*^22^, it blocked RCH responses in the flesh fly *Sarcophaga crassipalpis* and *Drosophila melanogaster*^23^,^24^. It is therefore clear that the partial pressure of oxygen (P_O2_) may interact with low temperature stress responses - affecting both benign and inducible forms of several traits associated with cold stress resistance - in at least some terrestrial insect species.

The biochemical mechanisms underlying oxygen and low temperature effects for insects, and especially the interactions there of, have not been well explored to date^25^,^26^,^27^. Since whole-animal metabolic rate is likely to be affected by both the aforementioned stressors, and may also determine the influence of these stressors, a metabolomics approach can provide insight into changes in metabolic pathways that may underlie oxygen and temperature stress responses. Previous research has shown the potential value of metabolomic profiling for investigating RCH or acclimation responses and cold shock. Such studies have yielded insights into the dynamic changes associated with cold tolerance by providing correlations between temperature tolerance or rates of recovery from chilling, and specific metabolites and key biochemical energy pathways^28^,^29^,^30^,^31^. Upregulation of anaerobic metabolites at low temperatures could explain some of the oxygen-temperature interactions, yet a comprehensive suite of metabolites have not been investigated at low temperatures under varying experimental oxygen levels^15^ (for anaerobic metabolism under hypoxia and heat stress, see)^14^. MacMillan *et al.*^32^ targeted some anaerobic metabolites, including alanine, and found no evidence of cold-induced anaerobic metabolism in *Gryllus pennsylvanicus*, while Michaud *et al.*^33^ found increased concentrations of alanine, glycerol and succinate in *Belgica antarctica* exposed to −10 °C, suggesting a switch to anaerobic metabolic pathways. However, there is little consensus on the mechanisms at play during cold and hypoxia stressors. The role of OCLTT in setting low temperature limits in terrestrial insects has not been well examined to date as most studies have focused on high temperature responses, and then typically only examined whole-animal metabolic rates (e.g.^34^,^35^, but see^13^,^32^). Results from the beetle *Tenebrio molitor* or cricket *Gryllus pennsylvanicus*^13^,^32^ suggest that CT_min_ is unlikely to be oxygen limited. However, with only these two studies available covering two distinct taxa, further research is essential, especially given the fundamental importance of this question.

To investigate the hypothesis that whole-animal oxygen limitation may set low temperature tolerance of insects we systematically investigated the influence of oxygen availability on several lethal and non-lethal metrics of low temperature tolerance. Here we make use of final instar larvae of a chill susceptible^36^ insect pest (false codling moth *Thaumatotibia leucotreta* (Meyrick) (Lepidoptera, Tortricidae)). Although the supercooling point (SCP) is not necessarily a useful measure of low temperature tolerance as its association with mortality depends on the species’ freeze tolerance strategy, in *T. leucotreta* larval SCP is equivalent to mortality temperatures^36^. Larvae of *T. leucotreta* are chill-susceptible with a CT_min_ of *c*. 6.7 °C when fed and a SCP of *c*. −15.6 °C under standard, benign laboratory rearing conditions^36^.

We determined the cold stress responses which are more frequently the focus of investigation in such studies by using thermolimit respirometry^37^ to determine CT_min_ under different controlled P_O2_. We estimated the critical oxygen partial pressure (P_crit_) for maintenance of whole-animal metabolic rate, and investigated changes in metabolites before, during and after chill coma. If chill coma endpoints are driven by oxygen availability, one major expectation is that hypoxia will increase CT_min_ (=less cold tolerant) while hyperoxia (increased oxygen) would decrease CT_min_ (=more cold tolerant)^13^,^15^ – relative to normoxia. We also measured SCP under different P_O2_ to assess if oxygen availability could influence this lethal estimate of low temperature tolerance. One prediction that can be made is that SCP would decrease under hypoxia if increased respiratory water loss, and the subsequent decrease in body water content, caused an increased concentration of solutes including cryoprotective molecules, while hyperoxia would be expected to have the opposite effect. In addition, OCLTT hypothesis predicts that if oxygen limitation is a primary driver of this functional low temperature performance limit, anaerobic metabolites should accumulate prior to CT_min_ under normoxia and hypoxia, but this effect should be relieved during hyperoxia. To examine this, 43 metabolites were measured across our suite of experimental conditions to test whether *T. leucotreta* likely employ anaerobic metabolism with the expectation that typical anaerobic metabolites such as lactic acid and alanine should be upregulated after hypoxic and potentially also low temperature exposures, but not hyperoxia. Furthermore, we predicted stronger anaerobic metabolite responses below, rather than above, P_crit_ levels, with associated concomitant changes in low temperature tolerance more pronounced under conditions further from homeostasis setpoints.

## Results

### Critical thermal minimum

Thermolimit respirometry (TLR)^37^ was used to determine critical thermal minima (CT_min_)^13^ under six different controlled P_O2_ conditions (2.5, 5, 10, 21, 40 kPa O_2_). Using a flow-through respirometry setup, individual larvae were cooled from 15 °C to −15 °C at a cooling rate of 0.25 °C min^−1^. The rate of CO_2_ release (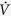
CO_2_) and activity data were analysed to determine CT_min_ (referred to as 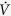
CO_2_ CT_min_ and activity CT_min_ respectively) following methods outlined in Klok *et al.*^12^ and Stevens *et al.*^13^. 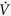
CO_2_ CT_min_ and activity CT_min_ were not significantly different from one another (n = 12 vs. n = 9; *Z* = 0.57, *P* = 0.57). The CT_min_ scored visually by an observer in a previous study^36^ was not significantly different from 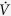
CO_2_ CT_min_ at 21 kPa O_2_ (n = 19 vs. n = 12; *Z* = 1.68, *P* = 0.09), but was different from activity CT_min_ (n = 19 vs. n = 9; *Z* = 3.25, *P* = 0.001) (Table 1). P_O2_ had no effect on 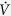
CO_2_ CT_min_ nor activity CT_min_, although activity CT_min_ could not be detected at 0 kPa O_2_ (*F*_5,56_ = 0.87, *P* = 0.51 and *H*_4,37_ = 3.48, *P* = 0.48; Table 1). Additional TLR parameters commonly measured are presented in online Supplementary materials (Table S1).

### Supercooling point

Supercooling points (SCP) under the six different P_O2_ were determined following a modified version of Boardman *et al.*^36^. SCP, which is lethal for *T. leucotreta*, was not affected by changes in P_O2_ (*F*_4,216_ = 0.65, *P* = 0.63, mass covariate = 39.29 mg, Fig. S1A), and start mass had a larger effect on SCP than P_O2_ (*P* = 0.01 vs. *P* = 0.63).

### Critical oxygen partial pressure and spiracle activity

**Figure 1:**
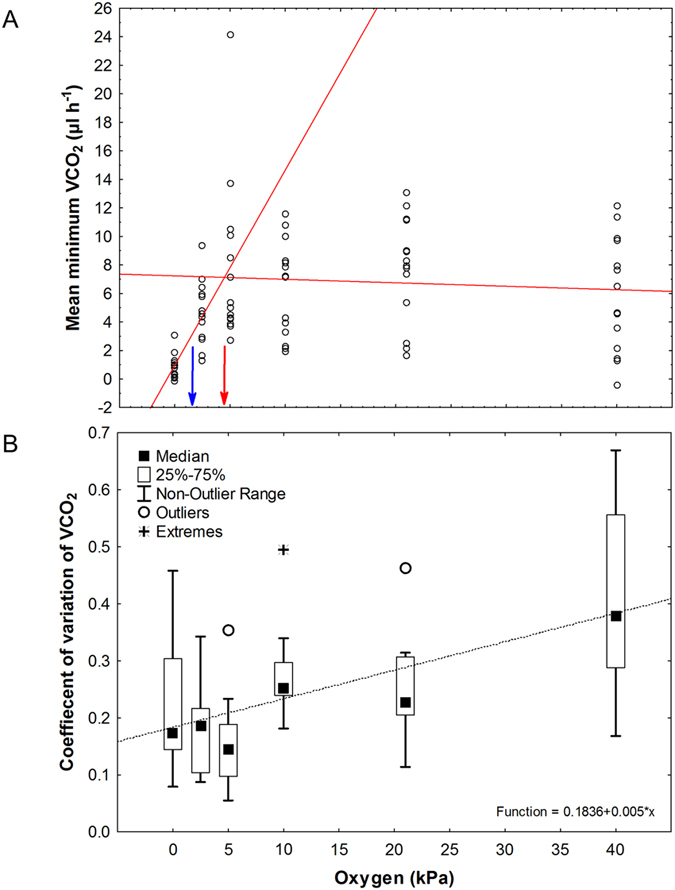
Critical oxygen partial pressure (P_crit_) (**A**) and coefficient of variation of 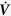
CO_2_ (COV) (**B**) for *T. leucotreta* larvae was determined under a range of oxygen conditions at 15 °C. All 14 individuals were recorded at all six O_2_ conditions. Raw mean 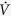
CO_2_ values are shown (A) and were used to calculate P_crit_. The blue and red lines and arrows indicate P_crit_ as estimated from regression tree analysis and linear regressions respectively (see methods for details). Box and whisker plots of the COV data (**B**) show the general trend for a decrease in COV as P_O2_ decreases (regression: r  = 0.55, *P* < 0.00001).

Multiplexed respirometry was used to determine the critical oxygen partial pressure (P_crit_) following Basson and Terblanche^38^ (further details^39^). Metabolic rate (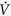
CO_2_) was determined for fourteen larvae at six experimental oxygen concentrations (2.5, 5, 10, 21, 40 kPa O_2_). P_crit_ was determined to be 4.5 kPa (Fig. 1A, full details on methodology in Supplementary materials). To better understand if active ventilation augmented gas exchange at rest, we estimated the degree of spiracle activity by calculating the coefficient of variation of 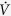
CO_2_ (COV)^40^. P_O2_ influenced spiracle behaviour as evidenced by 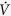
CO_2_ respirometry traces (Fig. S2). Under normoxia (21 kPa), as the temperature decreased, metabolic rate and spiracle activity gradually decreased until spiracle activity ceased at 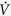
CO_2_ CT_min_ (Fig. S2B). As P_O2_ decreased, gas exchange patterns changed, spiracles remained open for longer periods and COV decreased (Fig. 1B, Friedman ANOVA: Χ^2^ = 39.14, df = 1, *P* < 0.0001; regression: r = 0.55, *P* < 0.00001).

### Metabolomic profiling

**Figure 2:**
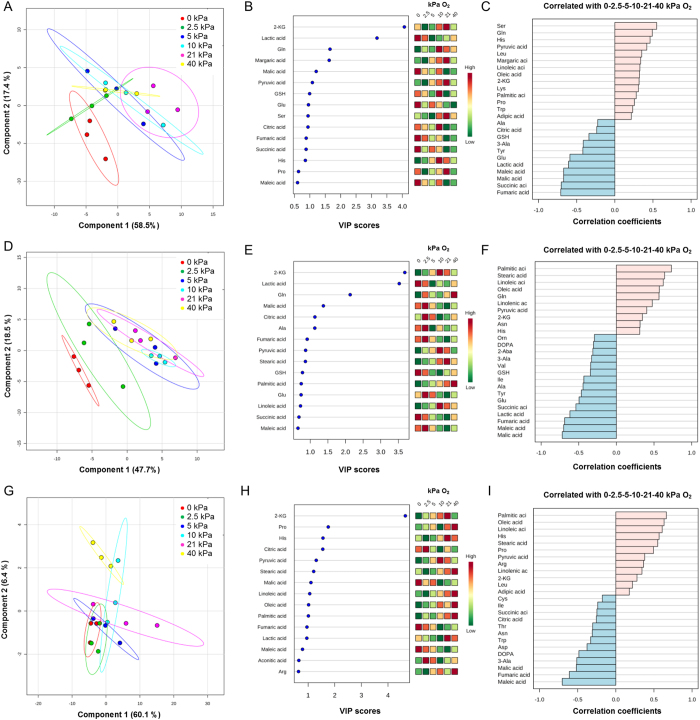
Results of metabolic profiling at each of the three timepoints sampled: before- (top row), during- (middle row) and after (bottom row) chill coma. 2D score plots (**A**,**D**,**G**) showing the projection of samples onto the first discriminant plane for each of the three timepoints. Each point is a sample (four individuals pooled), colours represent different oxygen treatments (red: 0 kPa O_2_, green: 2.5 kPa O_2_, blue: 5 kPa O_2_, turquoise: 10 kPa O_2_, pink: 21 kPa O_2_, yellow: 40 kPa O_2_) and lines indicate 95% confidence intervals. The variable importance plots (**B**,**E**,**H**), based on VIP scores, shows the top 15 variables that contribute to component 1 at each of the timepoints (based on Pearson correlation). The coloured boxes on the right indicate the relative concentrations of each of the metabolites in each oxygen treatment, with reds indicating highest concentrations and greens the lowest concentrations. Pattern finding analysis (**C**,**F**,**I**) at each timepoint sampled shows the top 25 metabolites that correlated with the increase in oxygen treatments (i.e. from 0 to 40 kPa O_2_). Metabolites in red are positively correlated with the increase in oxygen (i.e. highest concentrations at highest oxygen), while those in blue were negatively correlated (i.e. highest concentrations at lowest oxygen). Note that these results do not necessarily denote significance. Significant differences between oxygen treatments within each timepoint can be found in Table S3. Refer to Table S2 for compound abbreviations.

In order to investigate the metabolic changes in *T. leucotreta* larvae under the different gas conditions and at different timepoints surrounding chill coma, we measured changes in metabolites following Koštál *et al.*^41^. Samples for metabolomics were obtained by repeating the thermolimit respirometry (TLR) experiment with minor modifications under each of the six gas conditions. The modifications were that after the initial 15 °C for 30 min (“before chill coma”) and cooling to −2 °C at a ramp rate of 0.25 °C min^−1^, insects were held at −2 °C for 30 min (“during chill coma”). Thereafter, insects were immediately returned to 15 °C and allowed 2 h to recover (“after chill coma”). The forty-three metabolites detected in *T. leucotreta* included 26 amino acids and peptides, 7 tricarboxylic (TCA) cycle metabolites, 9 other organic acids and a biogenic amine (Table 2). There were clear effects of both temperature and oxygen, as well as significant interaction effects between temperature and oxygen, on the concentration of most metabolites in *T. leucotreta* (Table S2, Fig. 2). As we wanted to identify metabolite changes that were attributed to the effects of chill coma (“low temperature effects”) and to the oxygen levels before, during or after chill coma (“oxygen effects… chill coma”), results are presented in these respective sections below. Additional data exploring the temporal changes during chill coma under each oxygen concentration are presented in the online supplementary results and Figures.

#### Low temperature effects

Several metabolites varied under normoxia (21 kPa O_2_) at different timepoints before, during and after chill coma (Fig. S4C). As the timepoints overlapped in the Partial-Least Squares Discriminant Analysis (PLS-DA), neither component 1, that contributed 63.2% of the variance) nor component 2 (17.9% variance) could be used to clearly separate them (Fig. S4C, permutation test, *P* = 0.516). Variance of importance (VIP) scores indicated that component 1 was loaded by 2-ketoglutaric acid, lactic acid and margaric acid (Fig. S4D). 2-ketoglutaric acid and margaric acid were not significantly different between timepoints (Table 2, Fig. S7, Fig. S8). Lactic acid was significantly increased relative to the control (=before chill coma) after recovery from chill coma (*P* < 0.0037, Table 2, Fig. S8).

#### Oxygen effects before chill coma

After 30 min at 15 °C, before cooling and undergoing chill coma, the PLS-DA separated the data into two groups (permutation test, *P* = 0.028, Fig. 2A). Anoxia was considerably different from P_O2_ groups >5 kPa O_2_, with 2.5 kPa samples falling as intermediates. Component 1 contributed 58.5% of the variance and this component was loaded chiefly by 2-ketoglutaric and lactic acids, shown by the VIP scores (Fig. 2B). 2-ketoglutaric acid was highest in normoxia and decreased under altered oxygen (Fig. 2B, Fig. S7), with significant differences between 21 kPa and 5 kPa O_2_ (Table S3). Lactic acid was significantly elevated in anoxia (Table S3, Fig. 2B, Fig. S8). Other metabolites that showed a positive correlation with oxygen levels include serine, glutamine, histidine and pyruvic acid; while increases in lactic, maleic, malic, succinic and fumaric acids were correlated with a decrease in oxygen availability (Fig. 2C).

#### Oxygen effects during chill coma

After 2 h in chill coma at 0 °C, the data could be separated by PLS-DA into 3 groups: anoxia, 2.5 kPa O_2_ and then the rest of the oxygen treatments (Fig. 2D, permutation test, *P* < 0.001). As in the earlier results from “oxygen effects before chill coma”, changes in 2-ketoglutaric and lactic acids were the driving forces behind component 1 (47.7%) that separated the three groups, together with glutamine, malic acid, citric acid and alanine (Fig. 2E, Fig. S5, Fig. S7). Increases in palmitic, stearic, linoleic and oleic fatty acids and glutamine were all positively correlated with oxygen levels; increases in lactic, fumaric, maleic and malic acids were negatively correlated with oxygen availability during chill coma (Fig. 2F, Figs S5–S8).

#### Oxygen effects after chill coma

After 2 h recovery from chill coma, the oxygen treatments all clustered together (Fig. 2G, permutation test, *P* = 0.132). 2-ketoglutaric acid remained the main contributor to component 1 (60.1%), followed by proline, histidine and citric acid. 2-ketoglutaric acid, proline and histidine were reduced under hypoxia, while citric acid was elevated under hypoxia (Fig. 2H, Fig. S5, Fig. S7). After recovery, palmitic, oleic and linoleic fatty acids all increased with an increase in oxygen; while levels of maleic, fumaric and malic acids, together with 3-alanine were negatively correlated with oxygen availability (Fig. 2I, Figs S5–S8).

#### Pathway analysis

Pathway analyses on data from before chill coma and after chill coma were not significant after adjusting for multiple testing. Seven metabolic pathways (matched to known *Drosophila melanogaster* pathways) were significantly altered by oxygen availability during chill coma (Holm’s adjusted *P* < 0.05, Table 3). Significant pathways with high impact (i.e. changes occurring in more important nodes in the network will have a higher impact on the pathway) included three amino acid pathways (glycine, serine and threonine metabolism, arginine and proline metabolism and cysteine and methionine metabolism), glutathione metabolism and aminoacyl-tRNA biosynthesis (a key component in translation). Nitrogen metabolism and cyanoamino acid metabolism were also significantly enriched, but had zero impact, indicating that the changes occurred in marginal or relatively isolated positions in the pathway.

## Discussion

Three results of these experiments are most significant. First, we show here that *T. leucotreta* larvae are likely not oxygen limited at low temperatures or during chill coma, as exposure to different P_O2_ levels does not influence their low temperature tolerance scored as either activity limits (CT_min_) or lethal (SCP) limits. This result is largely in keeping with the handful of other studies of tracheate arthropods that have investigated low temperature responses under altered oxygen levels^13^,^32^. Furthermore, *T. leucotreta* appears capable of extracting sufficient oxygen to sustain aerobic metabolism from acutely hypoxic environments through careful regulation of their spiracles, excretory water loss and, by association, likely also ion regulation mechanisms. Finally, *T. leucotreta* clearly have some scope for anaerobic metabolism, which is likely to be of interest for post-harvest control of this pest when attempting to augment low temperature commodity disinfestation with modified atmospheres^42^,^43^, and may contribute to their ability to survive in fruits in which they routinely experience hypoxic and/or hypercapnic microenvironments. For example, internal oxygen concentrations of down to 1% have been documented in ‘Hass’ avocado fruit^44^, a known host of *T. leucotreta*^45^.

Responses of animals to hypoxia can be broadly classified as either the regulating class or the conforming class^46^,^47^. While the regulating class increases glycolytic flux in order to maintain normal processes during hypoxia, those in the conforming class decrease respiration rate, energy and substrate usage. Species that fall into the regulating class are typically able to maintain damage repair processes during hypoxia, but organisms in the conforming class will only start repair processes upon return to normoxia and usually survive long-term low oxygen exposures better than those in the regulating class. As P_O2_ decreases, typically at values close to P_crit_, animals switch from being oxy-regulators to being oxy-conformers^48^,^49^. *Thaumatotibia leucotreta* larvae likely follow this pattern, regulating normal processes above P_crit_, and switching to be oxy-conformers below P_crit_. The results of the metabolite profiling here further support this notion given that *T. leucotreta* larvae possess at least some ability to generate anaerobic metabolism end-products. This scope for anaerobic metabolism may be typical for larvae of holometabolous insects that routinely experience hypoxic conditions in artificial rearing mediums, necrotic fruit, or while wandering underground to pupate^6^.

We estimated P_crit_ of *T. leucotreta* to be *c*. 4.5 kPa, similar to other Lepidopteran larvae^50^,^51^, since this was the level at which metabolism was maintained before suppression at lower P_O2_ levels^52^. However, P_crit_ is notoriously variable, depending on a suite of methodological factors^38^,^53^,^54^ and on which metabolic parameter (resting or minimum) is employed, and this was evident in our results too (e.g. P_crit_ ranged from 1.25 to 6 kPa O_2_ at 15 °C). One curious result, suggesting that larvae actively enhance gas exchange at rest under even lower levels of P_O2_, is that at 2.5 kPa, a value much lower than our estimated P_crit_, larvae likely employed active gas exchange to boost metabolism, or perhaps may be reflective of a second critical P_O2_ value showing the onset of anaerobic metabolism reported for marine organisms^49^ (Table S1). Such increases in 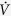
CO_2_ under hypoxia have also been documented in *Drosophila* at 3 kPa^55^ and scarabaeid beetles^56^ and has been attributed to either the stimulation of escape behaviour or an increase in tracheal conductance by increased ventilation or tidal volume (i.e. a switch to more convective gas exchange; see discussions in^40^,^56^,^57^). As activity CT_min_ at 2.5 kPa O_2_ are not significantly different to that estimated at 21 kPa O_2_, the nature of this increase in metabolic rate under hypoxia likely reflects a change in mode or pattern of gas exchange (see also Fig. S2).

Under hypoxia, insects typically modify spiracle behaviour (e.g. opening/closing frequency, duration) in order to maintain cellular respiration, for example by keeping their spiracles open for longer to ensure sufficient oxygen supply to metabolically-active tissues^17^,^58^. However, this may result in elevated respiratory water loss rates^18^,^19^,^20^, cellular oxidative damage^17^, and perturbation of haemolymph pH balance^59^. Under normoxia, *T. leucotreta* larvae actively excrete water (likely from their mid-gut, rather than intracellular fluid) in preparation for lower temperatures^36^,^39^. Regulation of water may partly explain why larvae appear to limit the number of excretion events during chilling exposure under hypoxia, but not other P_O2_ levels (Fig. S3). Regulation of excretion events may reflect a specific adaptation to remove potential ice nucleators from the gut^60^ or to control ion homeostasis and indirectly regulate low temperature freezing damage and facilitate rapid chilling recovery^61^. However, regulation of water flux at the whole-animal level is likely not a priority at values >P_crit_, but may be important near P_crit_ where a significant reduction in the number of excretion events was observed (Χ^2^ = 3.96, df = 1, *P* = 0.046, additional online results, Fig. S3). Body water content was not measured before and after cooling under different P_O2_ in this study as previous research has already shown that exposure to hypoxia decreases *T. leucotreta* body water content^27^.

CT_min_ remained unaffected under the different experimental P_O2_ conditions. These results therefore do not match the general expectation that CT_min_ would increase under hypoxia and decrease during hyperoxia, but is a similar outcome to what has been reported in *Tenebrio molitor* beetles^13^. In addition, SCP was unaffected by variation in P_O2_. Therefore, the chill tolerance of *T. leucotreta* larvae are unlikely to be oxygen limited at low temperatures since exposure to P_O2_ did not alter their lower limits to activity (i.e. CT_min_), nor the low temperature body freezing point (SCP). Our hypothesis regarding P_O2_ affecting SCP relied on significant changes in body water content. However, larvae survive up to 10 days without access to food and water^39^ suggesting considerable desiccation and starvation tolerance, perhaps limiting such a possible outcome under the present experimental conditions.

The metabolomic profiling reported here revealed that 2-ketoglutaric acid was the main metabolite measured in *T. leucotreta* larvae at all timepoints and treatments. This metabolite is an important intermediate in the Krebs cycle, and may also play a role as an antioxidant, much like histidine. Increased histidine has previously been associated with cold tolerance in *Drosophila*^31^,^62^. We also found that an increase in histidine was associated with an increase in oxygen availability (Table S3, Fig. 2C, Fig. S5), indicating that histidine may be an important antioxidant for scavenging reactive oxygen species (ROS) produced under hyperoxia^63^. The decrease in histidine under hypoxia may indicate the utilization of histidine to generate 2-ketoglutarate during oxidative stress, without engaging the tricarboxylic cycle^64^. Further evidence for this pathway may be found by the increase in succinic acid during hypoxia (Table S3, Fig. S7), as succinate is a by-product of the reaction of 2-ketoglutarate and ROS^64^. In summary, these results could indicate that these insects have a hypoxia-induced reactive oxygen species response, likely to modulate hypoxia-inducible factors (HIF) pathways^65^, and would be a worthwhile avenue for future research.

Metabolomic results from within each oxygen treatment showed that lower temperatures under normoxia or experimentally-decreased oxygen treatments are likely associated with an increased reliance upon anaerobic metabolism, as indicated by elevated lactic acid (Fig. S8). During hyperoxia, chilling these insects also resulted in increases in alanine, indicating that oxygen availability is likely not the main factor behind the switch to anaerobic metabolism (Table 2, Fig. S5). Another possibility is that mitochondrial function is strongly inhibited during chill coma, thus any effect of oxygen limitation on chill coma may be inconsequential for insects. Our data do however suggest some decoupling between glycolysis (running) and TCA (blocked) at low temperatures, but the cause is not clear. Additional work on tissue-specific metabolite responses would perhaps resolve this.

Within the timepoints measured (before, during, and after chill coma), changes in oxygen availability have diverse effects. Before chill coma (at 15 °C), greater oxygen availability results in higher concentrations of amino acids and pyruvic acid, indicating higher rates of aerobic metabolism. (see Fig. S2). During chill coma and after recovery, greater oxygen availability may allow for changes in fatty acid composition, which is likely useful for coping with subsequent temperature stress. Changes in lipid composition have a known association with chill coma recovery^66^,^67^,^68^, CT_min_^69^ and have recently been implicated in *T. leucotreta* low temperature tolerance^27^. In all cases, increased anaerobic metabolite concentrations with declining oxygen availability (especially lactic acid and alanine) indicates an increased reliance upon anaerobic metabolism, and a build-up of citric acid cycle intermediates indicates that the aerobic pathway is not working efficiently. 3-alanine, which is increased during hypoxia and cold in our study organism, can depress the electrical activity of the nerve chain in insects^70^ which could thus play a role in the immobility of insects in chill coma and under hypoxia.

The pathway analysis indicates that amino acid metabolism is significantly altered by oxygen availability during chill coma, both at translational and metabolic level (Table 3). The pathway with the highest impact was glycine, serine and threonine metabolism, which is overall reduced under hypoxia. Pyruvate generated by this pathway enters the citric acid cycle to produce adenosine triphosphate (ATP). Our data clearly shows a reduction in pyruvic acid under chill coma and all hypoxia treatments (Fig. S6) showing a lack of pyruvic acid available for aerobic metabolism. In addition, glutathione metabolism pathways are significantly increased during chill coma under hypoxia, leading to a significant increase in glutathione (Table 2, Fig. S6). Glutathione has antioxidant properties and has been well studied in association with cold tolerance in insects^71^. Further work from a diverse range of insect taxa is urgently required to address the generality of these results, but consensus thus far suggests insect low temperature tolerance traits are not governed by oxygen limitation despite potential induction of anaerobic metabolic pathways.

## Methods

### Insects

False codling moth *Thaumatotibia leucotreta* (Lepidoptera: Tortricidae) larvae were obtained from the Cedar Biocontrol Insectary, XSIT (Pty) Ltd, Citrusdal, South Africa and reared at 25 ± 5 °C at 50% relative humidity (L:D 12:12 h), under standard culture conditions (reviewed in Carpenter *et al.*)^72^. Final instar larvae were used in all experiments.

### Determination of cold tolerance under different oxygen conditions: Thermolimit respirometry

Thermolimit respirometry (TLR)^37^ was used to determine critical thermal minima (CT_min_) under different controlled P_O2_ conditions^16^. Different P_O2_ (0, 2.5, 5, 10, 40 kPa O_2_) were obtained from compressed cylinders with balance nitrogen (Air Products, South Africa), while normoxic air (21 kPa O_2_) was generated using an aquarium pump. All air was passed through a set of scrubber columns containing soda lime and 50:50 silica gel:Drierite (WA Hammond Drierite Company Ltd., Ohio, USA) to remove CO_2_ and H_2_O from the airstream. Rate of CO_2_ release (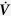
CO_2_) was measured in parts per million (ppm) using a calibrated Li-7000 infra-red gas analyser and data were recorded with standard LiCor software (LiCor, Lincoln, Nebraska, USA) on a desktop PC. Flow rate was maintained at 200 ml min^−1^ (STPD) using a mass flow control valve (Sidetrak, Sierra International, USA) connected to a mass flow control box (Sable Systems, Las Vegas, Nevada, USA) and activity was monitored electronically (AD-2, Sable Systems) in only a subset of individuals due to equipment constraints. Individual larvae were weighed before and after each respirometry run on a microbalance (accuracy ± 0.1 mg; AB104-S/Fact, Mettler Toledo International, Inc.).

Individual larvae were recorded separately in 2 mL cuvettes, and cuvettes were submerged in a programmable circulating and refrigeration bath filled with ethanol (CC410wl, Huber, Germany) set to follow a CT_min_ program: hold at 15 °C for 30 min to allow larvae within respirometry cuvettes to equilibrate with bath temperature; cool down to −15 °C at a ramp rate of 0.25 °C min^−1^. Temperature logging with iButtons (DS1922L, accuracy ± 0.5 °C, Dallas Semiconductors, Dallas, Texas, USA) revealed that the average cooling rate achieved by the bath during TLR recordings was ~0.24 °C min^−1^. This was repeated for n = 8 to 14 larvae for each P_O2_ (0, 2.5, 5, 10, 21, 40 kPa O_2_). No individuals were re-used at another oxygen level.

### Supercooling point (SCP) under different oxygen conditions

Supercooling points under different P_O2_ were determined following a modified version of Boardman *et al.*^36^. Pre-weighed larvae were placed in a 0.5 ml microtube (with air holes) in contact with a thermocouple (T-type, 0.005 m gauge, Omega Engineering, Inc., Stamford, CT). Larvae were placed in an airtight 0.6 L container, plumbed to receive air from either an aquarium pump (21 kPa O_2_), or a pressurised cylinder (0, 2.5, 10, 21, 40 kPa O_2_). The container was submerged in the programmable bath, set to follow the same temperature program as for TLR, except that cooling was continued down to −30 °C. Insect body temperatures were recorded at 1 Hz using a USB TC-08 thermocouple datalogger (Pico Technology, UK) connected to a desktop computer. The SCP was detected as the temperature just prior to the release of latent heat of crystallization^73^. A total of n = 32 or n = 48 larvae were analysed under each P_O2_. SCP under 21 kPa O_2_ was repeated with both air directly from the aquarium pump and air scrubbed of CO_2_ and H_2_O (see respirometry setup above) in order to ensure no differences were obtained as a result of the source. Data were pooled as there was no significant difference between the scrubbed or unscrubbed air (t_46_ = −0.003, *P* = 0.99).

### Determination of critical oxygen partial pressure (P_crit_)

Multiplexed respirometry was used to determine the critical oxygen partial pressure (P_crit_) following Basson and Terblanche^38^ (further details^39^). The basic respirometry setup was as described above for TLR, with the addition of a multiplexer (Sable Systems, RM8 Intelligent Multiplexer, V5) which allowed for the measurement of 7 individuals per respirometry run with 1 spare baseline channel.

Seven larvae at a time were placed in individual 2 mL cuvettes maintained at 15 °C using the programmable bath (total n = 14). For each P_O2_ treatment, gases were switched manually at the start of a 30 min baseline recording. Thereafter, each cuvette was measured for 30 min at each P_O2_ and the order for five oxygen concentrations (2.5, 5, 10, 21, 40 kPa O_2_) was randomised. In all cases however, the final exposure to 0 kPa O_2_ was performed last as the effects of anoxia on these larvae are unknown and to avoid any potential damaging effects of reperfusion injury^38^,^74^. Inactive channels were also flushed at 200 ml min^−1^ (~29 ml min^−1^ per channel) with the same gas as the active channel. All 14 individuals were therefore recorded at all six P_O2_ conditions. *T. leucotreta* larvae are able to survive at least 10 days at low (10%) relative humidity so the lengthy exposure to dry air was not considered to be problematic^39^. Baseline recordings were taken before and after each respirometry run to correct for potential analyser drift which was typically negligible.

### Respirometry data extraction and analyses

All respirometry files (TLR and Pcrit experiments) were baseline drift-corrected and 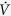
CO_2_ was converted to μl h^−1^ using Expedata version 1.8.2 (Sable Systems International).

For TLR, the 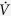
CO_2_ and activity data were analysed to determine 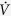
CO_2_ CTmin and activity CTmin following methods similar to Klok *et al.*^12^ and Lighton and Turner^37^ and expanded to CT_min_ usage in Stevens *et al.*^13^. Using custom-written automated data extraction scripts (macros), 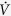
CO_2_ data were converted to absolute difference sum (ADS, the cumulative sum of absolute differences between adjacent measurements). The actual inflection point of the ADS was calculated by selecting the period of data around the inflection point, and fitting a linear regression through this selection. The highest residual from this linear regression corresponds to the time at which 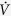
CO_2_ CT_min_ occurred. 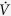
CO_2_ CT_min_ (in °C) was calculated using the observed cooling rate of 0.24 °C min^−1^.

Activity CT_min_ was calculated in a similar manner, with the exception that the data for the selection of the infection point was restricted to the portion of the file that corresponded to the end of activity (determined by visual inspection). Activity data were not adequate for determining CT_min_ under anoxic conditions as the gas causes immobility of the larvae within 30 min (i.e. before cooling starts).

Thereafter, average metabolic rate (MetAve) and minimum metabolic rate (MetMin) during cooling (from 15 °C to 0 °C) were calculated. The temperature at which MetMin occurred (TMetMin) was calculated using the observed cooling rate of 0.24 °C min^−1^. In a small handful of files, certain variables were not able to calculated (see sample sizes in Table S1) and these variables were excluded from analyses.

For P_crit_, the central 20 min from each individual under each P_O2_ was targeted for analysis using custom written macros. In order to reduce the potential effects of activity on influencing P_crit_ estimates, within this central 20 min, the mean of the most level 300 s was extracted to represent “resting metabolic rate” and the mean of the lowest 30 s was extracted to represent “minimum metabolic rate”. In order to measure the degree of spiracle activity, the coefficient of variation of 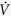
CO_2_ (COV) was calculated as the standard deviation divided by the mean 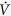
CO_2_^40^ from the central 20 min of each individual, under each P_O2_.

### Respirometry data analysis

Data were checked for normality and equal variance, and where these assumptions were violated, non-parametric tests were used. Where body size (i.e. fresh mass) was a significant covariate, an analysis of covariance (ANCOVA) was used with P_O2_ as the categorical variable and body mass as the continuous predictor variable. All statistics were performed using Statistica software (v.12, StatSoft, Inc., Tulsa, OK, USA) unless otherwise mentioned. Overlapping 95% confidence intervals were used to identify homogenous groups.

The 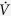
CO_2_ CT_min_ and activity CT_min_ were compared to data from Boardman *et al.*^36^ where CT_min_ was scored visually (referred to as “visual CT_min_”) using a non-parametric t-test. The 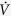
CO_2_ CT_min_ were compared between P_O2_ using an ANCOVA as mass was a significant correlate (r = −0.27, *P* = 0.04). Activity CT_min_, MetAve, MetMin and TMetMin were compared using Kruskall-Wallis ANOVA. Differences in excretion events during cooling were compared using a generalized linear model with a binomial distribution and logit link function. Overall, start mass was significantly correlated with SCP (r = 0.17, *P* = 0.01) therefore an ANCOVA was used to compare the effects of different P_O2_ on SCP.

P_crit_ data can be analysed using a variety of common methods: ordinary least square regression, piece-wise linear regression, non-linear regression, t-tests and one-way repeated measures Friedman ANOVA^38^,^53^,^54^. Here, we have used t-tests, ordinary least square regression and a novel method for determining P_crit_ using a boosted regression tree. Firstly, Wilcoxon matched pairs tests were used to compare between close P_O2_ groups (0 and 2.5 kPa, 2.5 and 5 kPa, etc) to identify the area in which the P_crit_ lies. Secondly, an ordinary least-squares regression was fitted to the 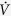
CO_2_ data from 0, 2.5 and 5 kPa O_2_, and a second regression was fitted to the 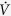
CO_2_ data from 10, 21 and 40 kPa O_2_. The intercept of the two equations for these regressions was considered P_crit_. Lastly, 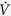
CO_2_ data were analysed using a boosted regression tree analysis with 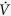
CO_2_ as the dependent variable and P_O2_ as the continuous variable in Statistica v. 12. The three methods were applied on both resting and minimum metabolic rate data, and generally produced values in close agreement.

### Metabolomic profiling

In order to investigate the metabolic changes in *T. leucoptreta* larvae under the different gas conditions and at different timepoints surrounding chill coma, we measured the change in metabolites following Koštál *et al.*^41^. Samples for profiling were obtained by repeating the thermolimit respirometry (TLR) experiment with minor modifications. Under each of the six gas conditions (0, 2.5, 5, 10, 21, 40 kPa O_2_), a modified CT_min_ program was followed: temperature was held at 15 °C for 30 min (“before chill coma”); cooled down to −2 °C at a ramp rate of 0.25 °C min^−1^ and held at −2 °C for 30 min (“during chill coma”) immediately returned to 15 °C and allowed 2 h to recover (“after chill coma”). Samples were obtained at all 3 timepoints. All samples were immediately frozen in liquid nitrogen and stored at −80 °C. Prior to extraction, individuals were thawed and weighed on a microbalance (accuracy ± 0.1 mg; AB104-S/Fact, Mettler Toledo International, Inc.) to determine fresh mass. Individual larvae were homogenised and metabolites were extracted in 70% ethanol^41^ and refrozen at −80 °C until analysis. Homogenates from four individuals were pooled for each sample. Three samples per oxygen and timepoint were analysed. Despite the potential instability of metabolites during the weighing and extraction process, only largely stable metabolites were included and all comparisons were made between samples within our own study subjected to identical procedures.

The metabolomic profiles were extensively investigated by a combination of GC/MS and liquid chromatography (LC) coupled to MS (LC/MS) in the ethanolic extracts after their treatment with ethyl chloroformate under pyridine catalysis and simultaneous extraction in chloroform^41^. The GC/MS acidic metabolite profiles were obtained on a VF-17 capillary column (Agilent, Santa Clara, CA, USA) coupled to a single quadrupole mass spectrometer (ISQ) (Thermo Scientific, San Jose, CA, USA) equipped with an electron impaction ion source and operated in the full-scan mode from 40 to 500 amu. A Trace 1300 gas chromatograph with TriPlus RSH autosampler (both from Thermo Scientific, San Jose, CA, USA), injector and interface holding at 280 °C, was directly coupled to the mass spectrometer via an interface held at 280 °C. An 1 μL aliquot of the chloroform extract was injected in the splitless mode into the GC/MS column. Oven temperature was initially maintained at 45 °C for 1.5 min. Thereafter, it was raised to 330 °C at a rate of 16 °C min^−1^ and maintained for 2 min. Helium was used as the carrier gas and delivered at a constant flow rate of 1.1 mL min^−1^.

LC/MS metabolite profiles were measured after evaporating a 30 μL aliquot of the chloroform extract to dryness by using a mild stream of nitrogen. After dissolution in 100 μL of the LC mobile phase, a 5 μL aliquot was injected into and separated on a Kinetex C18 column (150 × 3 mm; internal diameter [ID], 2.6 μm; Phenomenex, Torrance, CA, USA) at 35 °C at a flow rate of 400 μL min^−1^, using a gradient elution with the mobile phase consisting of (A) 5 mM ammonium formate in methanol and (B) 5 mM ammonium formate in water. The gradient elution program was linear from 30% to 100% A for 11 min, then held at 100% A for 1 min, and finally equilibrated for 4 min. The column eluent was directly introduced into a linear quadrupole ion trap mass spectrometer (LTQXL; Thermo Fisher, San Jose, USA) equipped with a HESI II electrospray ionization source operated at 3.5 kV and scanning mass range 150–850 Da.

The data were processed with the Thermo Scientific Xcalibur 2.1 software and an in-house developed Metabolite Mapper platform, which provides automated peak detection and metabolite deconvolution by employing retention time and mass spectral and detector response features, followed by time alignment of the data obtained in each particular analysis for a defined experimental sample set and generation of data matrix, which is automatically exported to a predefined Microsoft Excel™ spreadsheet for further statistical processing. The metabolites were identified against relevant standards and further subjected to quantitative analysis by using an internal standard calibration method. All chemicals used were purchased from Sigma-Aldrich Co. (St. Louis, MO, USA), except the isotope-labeled metabolites used as internal calibration standards, which were obtained from Cambridge Isotope Laboratories (Andover, MA, USA). Whole-body concentrations of the metabolites were recalculated as nmol mg^−1^ using pooled sample mass. While there is a potential bias in scaling the data using fresh mass as body water content may have been altered by the P_O2_ treatments, data were analysed using a more conservative non-parametric GLZ using a normal distribution and identity link function.

### Metabolomic data analysis

For each of the 43 metabolites detected, the effects of oxygen and timepoint were analysed independently with a GLZ using a normal distribution and identity link function (SAS 9.3, SAS Institute, Cary, NC, USA). Data were analysed both within timepoint to identify changes due solely to variation in oxygen levels and within an oxygen level to identify changes that are likely due to chill coma. Significance levels were adjusted for false discovery rate correction (FDR) using Benjamini-Hochberg procedure^75^. In all cases, FDR-corrected *P*-values were used to determine significance.

Multivariate analysis of whole-system metabolic changes among the oxygen treatments were conducted in MetaboAnalyst 2.0 and 3.0^76^,^77^ using Partial-Least Squares Discriminant Analysis (PLS-DA) following Colinet *et al.*^78^. The significance of the PLS-DA was determined using default permutation tests (1000 permutations, b/w distance separation) in MetaboAnalyst. Variance of importance score (VIP) and pattern finding analyses were used to identify changes in metabolites with VIP scores above 1.5 considered to be significant. Data were scaled using Pareto scaling (mean-centred and divided by the square root of the standard deviation of each variable) and each timepoint were analysed independently. Thereafter, pathway analysis combining pathway enrichment analysis with topology analysis using *Drosophila melanogaster* specific library was conducted on the entire dataset for each timepoint independently to identify enriched metabolic pathways, and the impact of these enrichments. Globaltest pathway enrichment method was used, and the node importance measure was relative betweenness centrality.

## Additional Information

**How to cite this article**: Boardman, L. *et al.* Cold tolerance is unaffected by oxygen availability despite changes in anaerobic metabolism. *Sci. Rep.* **6**, 32856; doi: 10.1038/srep32856 (2016).

## Supplementary Material

Supplementary Information

## Figures and Tables

**Table 1:** Critical thermal minima determined by thermolimit respirometry (TLR) under different P_O2_ conditions.

O_2_ (kPa)	Visual CT_min_* (°C)	Mass (mg)	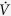 CO_2_ CT_min_ (°C)	Activity CT_min_ (°C)
0		38.69 ± 7.66(n = 8)	7.33 ± 2.08(n = 8)	Undetectable(n = 4)
2.5		50.25 ± 6.88(n = 11)	4.27 ± 0.78(n = 11)	4.54 ± 1.00(n = 9)
5		43.53 ± 9.37(n = 8)	3.82 ± 1.64(n = 6)	3.93 ± 1.28(n = 8)
10		44.84 ± 9.48(n = 14)	5.03 ± 0.49(n = 14)	7.09 ± 1.48(n = 6)
21	6.7 ± 0.1(n = 19)	40.31 ± 6.70(n = 13)	5.45 ± 0.53(n = 12)	5.10 ± 0.69(n = 9)
40		44.99 ± 10.87(n = 12)	4.65 ± 0.72(n = 12)	5.80 ± 1.20(n = 5)
		**F_4,50_ = 1.04,*P* = 0.40**	**F_5,56_ = 0.87,*P* = 0.51**	**H_4,37_ = 3.48,*P* = 0.48**

Data indicates mean  ±  S.E.M. Statistical results of comparison between oxygen groups are shown in the bottom row. Additional results in Table S1. 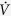
CO_2_ CT_min_ and activity CT_min_ were calculated from the inflection point of regressions of the absolute difference sum (ADS) residuals (see methods).

^*^Critical thermal minimum (CT_min_) data from Boardman *et al.*^36^.

**Table 2:** Summary of significant differences between timepoints (Time) within each oxygen treatment (O_2_).

Group1	O_2_	0	0	0	2.5	2.5	2.5	5	5	5	10	10	10	21	21	21	40	40	40
*vs*	Time	1	1	2	1	1	2	1	1	2	1	1	2	1	1	2	1	1	2
Group 2	O_2_	0	0	0	2.5	2.5	2.5	5	5	5	10	10	10	21	21	21	40	40	40
	Time	2	3	3	2	3	3	2	3	3	2	3	3	2	3	3	2	3	3
*Amino acids and peptides*																			
Ala					•	•		*		*							•	•	
Arg								•										•	
Asn			*	*		•		•	*								•	•	
Asp								*	•										
3-Ala						*		•	•										
Cys			•						*									•	
Glu								•	•							*	•	*	
Gln		•	•	*				•									•		
Gly								*	•										
His																			
Ile		•	•		•			•	•										
Leu		•	•		•	*			*									•	
Lys			•		*	*													
Met									•										
Orn						*	•												
Phe						*			*									*	
Pro													•					•	•
Ser				*				*	•	*				*					
Thr																			
Trp					•	•			•										
Tyr																			
Val					*	•		*	•									•	
2-Aminobutyric acid (2-Aba)								•											
Cystathionine (CTH)																			
3,4-Dihydroxyphenylalanine (DOPA)		•	*		•	*													
Glutathione (GSH)					*	•													
*TCA cycle metabolites*																			
Aconitic acid					•	*													
2-Ketoglutaric acid (2-KG)																			
Citric acid					•	*		•											
Fumaric acid					*		*				*	*							
Malic acid			•	•	*	*		*		*									
Pyruvic acid											•	•							
Succinic acid					•													•	
*Other organic acids*																			
Adipic acid																			
Lactic acid		*		*	•		•								•				
Maleic acid					*		*				*	*							
Linoleic acid																		*	
Linolenic acid									•									•	
Margaric acid																			
Oleic acid																		•	
Palmitic acid																		*	
Stearic acid																		*	
*Biogenic amine*																			
Putrescine								•					•						

Time 1 is before chill coma, time 2 is during chill coma and time 3 is after chill coma. Significant effects (*P* < 0.0037 after false discovery rate correction using the Benjamini-Hochberg procedure^75^) are indicated by symbols. The level of significance is indicated by the symbols: blank is *P* > 0.0037, dot (•) is 0.0037>*P* > 0.001, and star (*) is *P* < 0.001.

**Table 3:** Summary of the metabolic pathways that were altered by oxygen availability during chill coma.

Pathway	Number of hits (total cmpd)	Hits (KEGG ID)	Hits	*P*-value	Holm adjusted *P*-value	FDR	Impact
*Aminoacyl-tRNA biosynthesis* KEGG dme00970 Translation	19 (67)	C00025	L-Glutamic acid	0.000	0.006	0.004	0.138
		C00037	Glycine				
		C00041	L-Alanine				
		C00047	L-Lysine				
		C00049	L-Aspartic acid				
		C00062	L-Arginine				
		C00064	L-Glutamine				
		C00065	L-Serine				
		C00073	L-Methionine				
		C00078	L-Tryptophan				
		C00079	L-Phenylalanine				
		C00082	L-Tyrosine				
		C00123	L-Leucine				
		C00135	L-Histidine				
		C00148	L-Proline				
		C00152	L-Asparagine				
		C00183	L-Valine				
		C00188	L-Threonine				
		C00407	L-Isoleucine				
*Glutathione metabolism* KEGG dme00480 Metabolism of other amino acids	5 (26)	C00025	L-Glutamic acid	0.000	0.012	0.004	0.494
		C00037	Glycine				
		C00051	Glutathione				
		C00077	Ornithine				
		C00134	Putrescine				
*Glycine, serine and threonine metabolism* KEGG dme00260 Amino acid metabolism	5 (25)	C00022	Pyruvic acid	0.000	0.013	0.004	0.571
		C00037	Glycine				
		C00065	L-Serine				
		C00188	L-Threonine				
		C02291	L-Cystathione				
*Nitrogen metabolism* KEGG dme00910 Energy metabolism	4 (7)	C00025	L-Glutamic acid	0.001	0.031	0.006	0.000
		C00037	Glycine				
		C00064	L-Glutamine				
		C02291	L-Cystathionine				
*Cyanoamino acid metabolism* KEGG dme00460 Metabolism of other amino acids	2 (6)	C00037	Glycine	0.001	0.035	0.006	0.000
		C00065	L-Serine				
*Arginine and proline metabolism* KEGG dme00330 Amino acid metabolism	8 (37)	C00025	L-Glutamic acid	0.001	0.035	0.006	0.528
		C00049	L-Aspartic acid				
		C00062	L-Arginine				
		C00064	L-Glutamine				
		C00077	Ornithine				
		C00122	Fumaric acid				
		C00134	Putrescine				
		C00148	L-Proline				
*Cysteine and methionine metabolism* KEGG dme00270 Amino acid metabolism	4 (25)	C00022	Pyruvic acid	0.001	0.044	0.006	0.227
		C00065	L-Serine				
		C00073	L-Methionine				
		C02291	L-Cystathione				

Raw *P*-values are calculated from the enrichment analysis and significance is based on significant Holm-adjusted *P*-values, and the table is ranked by these values (only significant pathways are shown, *P* < 0.05). Pathway impact values are calculated from pathway topology analysis and indicate where changes in the metabolites are more likely to have a greater impact on the pathway based on the location of the metabolite within the pathway. FDR - false discovery rate.

## References

[b1] Chown S. L. & Nicolson S. W. Insect Physiological Ecology: mechanisms and patterns (Oxford University Press, 2004).

[b2] Harrison J. F., Woods H. A. & Roberts S. P. Ecological and Environmental Physiology of Insects (Oxford University Press, 2012).

[b3] Hoback W. W. & Stanley D. W. Insects in hypoxia. J. Insect Physiol. 47, 533–542 (2001). 11249941 10.1016/s0022-1910(00)00153-0

[b4] Dillon M. E. & Frazier M. R. *Drosophila melanogaster* locomotion in cold thin air. J. Exp. Biol. 209, 364–371 (2006). 16391358 10.1242/jeb.01999

[b5] Holter P. & Spangenberg A. Oxygen uptake in coprophilous beetles (*Aphodius*, *Geotrupes*, *Sphaeridium*) at low oxygen and high carbon dioxide concentrations. Physiol. Entomol. 22, 339–343 (1997).

[b6] Callier V., Hand S. C., Campbell J. B., Biddulph T. & Harrison J. F. Developmental changes in hypoxic exposure and responses to anoxia in *Drosophila melanogaster*. J. Exp. Biol. 218, 2927–2934 (2015). 26206351 10.1242/jeb.125849

[b7] Terblanche J. S. In The Insects - Structure and Function (eds Simpson S. J. & Douglas A. E.) 588–618 (2012).

[b8] Hazell S. P. & Bale J. S. Low temperature thresholds: Are chill coma and CTmin synonymous? J. Insect Physiol. 57, 1085–1089 (2011). 21510951 10.1016/j.jinsphys.2011.04.004

[b9] MacMillan H. A. & Sinclair B. J. Mechanisms underlying insect chill-coma. J. Insect Physiol. 57, 12–20 (2011). 20969872 10.1016/j.jinsphys.2010.10.004

[b10] Goller B. Y. F. & Esch H. Comparative study of chill-coma temperatures and muscle potentials in insect flight muscles. J. Exp. Biol. 150, 221–231 (1990).

[b11] Pörtner H. O. Climate change and temperature-dependent biogeography: oxygen limitation of thermal tolerance in animals. Naturwissenschaften 88, 137–146 (2001). 11480701 10.1007/s001140100216

[b12] Klok C. J., Sinclair B. J. & Chown S. L. Upper thermal tolerance and oxygen limitation in terrestrial arthropods. J. Exp. Biol. 207, 2361–2370 (2004). 15159440 10.1242/jeb.01023

[b13] Stevens M. M., Jackson S., Bester S. A., Terblanche J. S. & Chown S. L. Oxygen limitation and thermal tolerance in two terrestrial arthropod species. J. Exp. Biol. 213, 2209–2218 (2010). 20543119 10.1242/jeb.040170

[b14] Verberk W. C. E. P., Sommer U., Davidson R. L. & Viant M. R. Anaerobic metabolism at thermal extremes: a metabolomic test of the oxygen limitation hypothesis in an aquatic insect. Integr. Comp. Biol. 53, 609–619 (2013). 23604617 10.1093/icb/ict015 PMC3776598

[b15] Verberk W. C. E. P. *et al.* Does oxygen limit thermal tolerance in arthropods? A critical review of current evidence. Comp. Biochem. Physiol. Part A Mol. Integr. Physiol. 192, 64–78 (2016). 10.1016/j.cbpa.2015.10.020 PMC4717866 26506130

[b16] Boardman L. & Terblanche J. S. Oxygen safety margins set thermal limits in an insect model system. J. Exp. Biol. 218, 1677–1685 (2015). 26041031 10.1242/jeb.120261

[b17] Hetz S. K. & Bradley T. J. Insects breathe discontinuously to avoid oxygen toxicity. Nature 433, 516–519 (2005). 15690040 10.1038/nature03106

[b18] Terblanche J. S., Clusella-Trullas S. & Chown S. L. Phenotypic plasticity of gas exchange pattern and water loss in *Scarabaeus spretus* (Coleoptera: Scarabaeidae): deconstructing the basis for metabolic rate variation. J. Exp. Biol. 213, 2940–2949 (2010). 20709922 10.1242/jeb.041889

[b19] Williams C. M., Pelini S. L., Hellmann J. J. & Sinclair B. J. Intra-individual variation allows an explicit test of the hygric hypothesis for discontinuous gas exchange in insects. Biol. Lett. 6, 274–277 (2010). 19923135 10.1098/rsbl.2009.0803 PMC2865053

[b20] Huang S.-P., Talal S., Ayali A. & Gefen E. The effect of discontinuous gas exchange on respiratory water loss in grasshoppers (Orthoptera: Acrididae) varies across an aridity gradient. J. Exp. Biol. 218, 2510–2517 (2015). 26290590 10.1242/jeb.118141

[b21] Lee R. E., Chen C. P. & Denlinger D. L. A rapid cold-hardening process in insects. Science (80-) 238, 1415–1417 (1987). 10.1126/science.238.4832.1415 17800568

[b22] Coulson S. J. & Bale J. S. Anoxia induces rapid cold hardening in the housefly *Musca domestica* (Diptera: Muscidae). J. Insect Physiol. 37, 497–501 (1991).

[b23] Yocum G. D. & Denlinger D. L. Anoxia blocks thermotolerance and the induction of rapid cold hardening in the flesh fly, Sarcophaga crassipalpis. Physiol. Biochem. Zool. 19, 152– 158 (1994).

[b24] Nilson T. L., Sinclair B. J. & Roberts S. P. The effects of carbon dioxide anesthesia and anoxia on rapid cold-hardening and chill coma recovery in *Drosophila melanogaster*. J. Insect Physiol. 52, 1027–1033 (2006). 16996534 10.1016/j.jinsphys.2006.07.001 PMC2048540

[b25] Storey K. B. & Storey J. M. In Advances in Low Temperature Biology (ed Steponkus P. L.) 101–140 (JAI Press, 1992).

[b26] Boardman L., Sorensen J. G., Johnson S. A. & Terblanche J. S. Interactions between controlled atmospheres and low temperature tolerance: a review of biochemical mechanisms. Front. Physiol. 2, 92 (2011). 22144965 10.3389/fphys.2011.00092 PMC3228967

[b27] Boardman L., Sørensen J. G. & Terblanche J. S. Physiological and molecular mechanisms associated with cross tolerance between hypoxia and low temperature in *Thaumatotibia leucotreta*. J. Insect Physiol. 82, 75–84 (2015). 26376454 10.1016/j.jinsphys.2015.09.001

[b28] Michaud M. R. & Denlinger D. L. Shifts in the carbohydrate, polyol, and amino acid pools during rapid cold-hardening and diapause-associated cold-hardening in flesh flies (*Sarcophaga crassipalpis*): a metabolomic comparison. J. Comp. Physiol. B 177, 753–763 (2007). 17576567 10.1007/s00360-007-0172-5

[b29] Overgaard J. *et al.* Metabolomic profiling of rapid cold hardening and cold shock in *Drosophila melanogaster*. J. Insect Physiol. 53, 1218–1232 (2007). 17662301 10.1016/j.jinsphys.2007.06.012

[b30] Colinet H., Larvor V., Laparie M. & Renault D. Exploring the plastic response to cold acclimation through metabolomics. Funct. Ecol. 26, 711–722 (2012).

[b31] Williams C. M. *et al.* Cold adaptation shapes the robustness of metabolic networks in Drosophila melanogaster. Evolution (N. Y). 68, 3505–3523 (2014). 10.1111/evo.12541 PMC4472466 25308124

[b32] MacMillan H. A., Williams C. M., Staples J. F. & Sinclair B. J. Reestablishment of ion homeostasis during chill-coma recovery in the cricket *Gryllus pennsylvanicus*. Proc. Natl. Acad. Sci. 109, 20750–20755 (2012). 23184963 10.1073/pnas.1212788109 PMC3528563

[b33] Robert Michaud M. *et al.* Metabolomics reveals unique and shared metabolic changes in response to heat shock, freezing and desiccation in the Antarctic midge, Belgica antarctica. J. Insect Physiol. 54, 645–655 (2008). 18313070 10.1016/j.jinsphys.2008.01.003

[b34] Lighton J. R. B. Hot hypoxic flies: Whole-organism interactions between hypoxic and thermal stressors in *Drosophila melanogaster*. J. Therm. Biol. 32, 134–143 (2007).

[b35] Verberk W. C. E. P. & Bilton D. T. Can oxygen set thermal limits in an insect and drive gigantism? Plos One 6, e22610 (2011). 21818347 10.1371/journal.pone.0022610 PMC3144910

[b36] Boardman L., Grout T. G. & Terblanche J. S. False codling moth *Thaumatotibia leucotreta* (Lepidoptera, Tortricidae) larvae are chill-susceptible. Insect Sci. 19, 315–328 (2012).

[b37] Lighton J. R. B. & Turner R. J. Thermolimit respirometry: an objective assessment of critical thermal maxima in two sympatric desert harvester ants, *Pogonomyrmex rugosus* and *P. californicus*. J. Exp. Biol. 207, 1903–1913 (2004). 15107444 10.1242/jeb.00970

[b38] Basson C. H. & Terblanche J. S. Metabolic responses of *Glossina pallidipes* (Diptera: Glossinidae) puparia exposed to oxygen and temperature variation: Implications for population dynamics and subterranean life. J. Insect Physiol. 56, 1789–1797 (2010). 20673831 10.1016/j.jinsphys.2010.07.010

[b39] Boardman L., Sørensen J. G. & Terblanche J. S. Physiological responses to fluctuating thermal and hydration regimes in the chill susceptible insect, Thaumatotibia leucotreta. J. Insect Physiol. 59, 781–794 (2013). 23684741 10.1016/j.jinsphys.2013.05.005

[b40] Lighton J. R. B. & Lovegrove B. G. A temperature-induced switch from diffusive to convective ventilation in the honeybee. J. Exp. Biol. 154, 509–516 (1990).

[b41] Koštál V. *et al.* Long-Term cold acclimation extends survival time at 0°C and modifies the metabolomic profiles of the larvae of the fruit fly *Drosophila melanogaster*. Plos One 6, e25025 (2011). 21957472 10.1371/journal.pone.0025025 PMC3177886

[b42] Hallman G. J. & Denlinger D. L. Temperature sensitivity in insects and application in integrated pest management. (Westview Press, Inc., 1998).

[b43] Fields P. G. & White N. D. G. Alternatives to methyl bromide treatmerts for stored-product and quarantine insect. Annu. Rev. Entomol. 47, 331–359 (2002). 11729078 10.1146/annurev.ento.47.091201.145217

[b44] Ben-Yehoshua S., Robertson R. N. & Biale J. B. Respiration & internal atmosphere of avocado fruit. Plant Physiol. 38, 194–201 (1962). 16655774 10.1104/pp.38.2.194 PMC549905

[b45] Grové T., De Beer M. S. & Joubert P. H. Developing a systems approach for *Thaumatotibia leucotreta* (Lepidoptera: Tortricidae) on ‘Hass’ avocado in South Africa. J. Econ. Entomol. 103, 1112–1128 (2010). 20857718 10.1603/ec09045

[b46] Makarieva A. M., Gorshkov V. G., Li B.-L. & Chown S. L. Size- and temperature-independence of minimum life-supporting metabolic rates. Funct. Ecol. 20, 83–96 (2006).

[b47] Hochachka P. W. In Plant Life Under Oxygen Deprivation (eds Jackson M. B., Davies D. D. & Lambers H.) 121–128 (SPB Academic Publishing, 1991).

[b48] Yeager D. P. & Ultsch G. R. Physiological regulation and conformation: a BASIC program for the determination of critical points. Physiol. Zool. 62, 888–907 (1989).

[b49] Pörtner H. O. & Grieshaber, M K. In The vertebrate gas transport cascade: adaptations to environment and mode of life (ed. Bicudo J. E. P. W.) 330–357 (CRC Press, Boca Raton, Florida, 1993).

[b50] Greenlee K. J. & Harrison J. F. Respiratory changes throughout ontogeny in the tobacco hornworm caterpillar, Manduca sexta. J. Exp. Biol. 208, 1385–1392 (2005). 15781898 10.1242/jeb.01521

[b51] Zhou S., Criddle R. S. & Mitcham E. J. Metabolic response of Platynota stultana pupae during and after extended exposure to elevated CO_2_ and reduced O_2_ atmospheres. J. Insect Physiol. 47, 401–409 (2001). 11166305 10.1016/s0022-1910(00)00124-4

[b52] Herreid C. F. Hypoxia in invertebrates. Comp. Biochem. Physiol. Part A Physiol. 67, 311–320 (1980).

[b53] Clusella-Trullas S. & Chown S. L. Investigating onychophoran gas exchange and water balance as a means to inform current controversies in arthropod physiology. J. Exp. Biol. 211, 3139–3146 (2008). 18805813 10.1242/jeb.021907

[b54] Marshall D. J., Bode M. & White C. R. Estimating physiological tolerances - a comparison of traditional approaches to nonlinear regression techniques. J. Exp. Biol. 216, 2176–2182 (2013). 23470657 10.1242/jeb.085712

[b55] Van Voorhies W. A. Metabolic function in *Drosophila melanogaster* in response to hypoxia and pure oxygen. J. Exp. Biol. 212, 3132–3141 (2009). 19749106 10.1242/jeb.031179 PMC2742449

[b56] Lease H. M., Klok C. J., Kaiser A. & Harrison J. F. Body size is not critical for critical PO2 in scarabaeid and tenebrionid beetles. J. Exp. Biol. 215, 2524–2533 (2012). 22723492 10.1242/jeb.057141

[b57] Harrison J. *et al.* Responses of terrestrial insects to hypoxia or hyperoxia. Respir. Physiol. Neurobiol. 154, 4–17 (2006). 16595193 10.1016/j.resp.2006.02.008

[b58] Terblanche J. S., Marais E., Hetz S. K. & Chown S. L. Control of discontinuous gas exchange in *Samia cynthia*: effects of atmospheric oxygen, carbon dioxide and moisture. J. Exp. Biol. 211, 3272–3280 (2008). 18840661 10.1242/jeb.022467

[b59] Groenewald B., Chown S. L. & Terblanche J. S. A hierarchy of factors influence discontinuous gas exchange in the grasshopper *Paracinema tricolor* (Orthoptera: Acrididae). J. Exp. Biol. 217, 3407–3415 (2014). 25063854 10.1242/jeb.102814

[b60] Lee R. E., Costanzo J. P. & Mugnano J. A. Regulation of supercooling and ice nucleation in insects. Eur. J. Entomol. 93, 405–418 (1996).

[b61] MacMillan H. A. & Sinclair B. J. The role of the gut in insect chilling injury: cold-induced disruption of osmoregulation in the fall field cricket, Gryllus pennsylvanicus. J. Exp. Biol. 214, 726–734 (2011). 21307058 10.1242/jeb.051540

[b62] Malmendal A. *et al.* Metabolomic analysis of the selection response of *Drosophila melanogaster* to environmental stress: are there links to gene expression and phenotypic traits? Naturwissenschaften 100, 417–427 (2013). 23571708 10.1007/s00114-013-1040-7

[b63] Wade A. M. & Tucker H. N. Antioxidant characteristics of L-histidine. J. Nutr. Biochem. 9, 308–315 (1998).

[b64] Lemire J. *et al.* Histidine is a source of the antioxidant, alpha-ketoglutarate, in Pseudomonas fluorescens challenged by oxidative stress. Fems Microbiol. Lett. 309, 170–177 (2010). 20597986 10.1111/j.1574-6968.2010.02034.x

[b65] Görlach A. In Hypoxia and cancer (ed. Melillo M.) 65–90 (Springer Science + Business Media B.V., 2014).

[b66] Overgaard J., Sørensen J. G., Petersen S. O., Loeschcke V. & Holmstrup M. Changes in membrane lipid composition following rapid cold hardening in *Drosophila melanogaster*. J. Insect Physiol. 51, 1173–1182 (2005). 16112133 10.1016/j.jinsphys.2005.06.007

[b67] Goto S. G., Udaka H., Ueda C. & Katagiri C. Fatty acids of membrane phospholipids in *Drosophila melanogaster* lines showing rapid and slow recovery from chill coma. Biochem. Biophys. Res. Commun. 391, 1251–1254 (2010). 20006581 10.1016/j.bbrc.2009.12.053

[b68] Pujol-Lereis L. M., Fagali N. S., Rabossi A., Catalá Á. & Quesada-Allué L. A. Chill-coma recovery time, age and sex determine lipid profiles in *Ceratitis capitata* tissues. J. Insect Physiol. 87, 53–62 (2016). 26868723 10.1016/j.jinsphys.2016.02.002

[b69] Slotsbo S. *et al.* Tropical to subpolar gradient in phospholipid composition suggests adaptive tuning of biological membrane function in drosophilids. Funct. Ecol. 30, 759–768 (2016).

[b70] Vereshtchagin S. M., Sytinsky I. A. & Tyshchenko V. P. The effect of γ-aminobutyric acid and β-alanine on bioelectrical activity of nerve ganglia of the pine moth caterpillar (*Dendrolimus pini*). J. Insect Physiol. 6, 21–25 (1961).

[b71] Joanisse D. R. & Storey K. B. Oxidative stress and antioxidants in overwintering larvae of cold-hardy goldenrod gall insects. J. Exp. Biol. 199, 1483–1491 (1996). 9319381 10.1242/jeb.199.7.1483

[b72] Carpenter J., Bloem S. & Hofmeyr H. In Area-Wide Control of Insect Pests (eds Vreysen M., Robinson A. & Hendrichs J.) 351–359 (Springer Netherlands, 2007).

[b73] Sinclair B. J., Jaco Klok C., Scott M. B., Terblanche J. S. & Chown S. L. Diurnal variation in supercooling points of three species of Collembola from Cape Hallett, Antarctica. J. Insect Physiol. 49, 1049–1061 (2003). 14568583 10.1016/j.jinsphys.2003.08.002

[b74] Lighton J. R. B. & Schilman P. E. Oxygen reperfusion damage in an insect. Plos One 2, e1267 (2007). 18060061 10.1371/journal.pone.0001267 PMC2092388

[b75] Benjamini Y. & Hochberg Y. Controlling the False Discovery Rate: A Practical and Powerful Approach to Multiple Testing. J. R. Stat. Soc. Ser. B 57, 289–300 (1995).

[b76] Xia J., Mandal R., Sinelnikov I. V., Broadhurst D. & Wishart D. S. MetaboAnalyst 2.0-a comprehensive server for metabolomic data analysis. Nucleic Acids Res. 40, W127–W133 (2012). 22553367 10.1093/nar/gks374 PMC3394314

[b77] Xia J., Sinelnikov I. V., Han B. & Wishart D. S. MetaboAnalyst 3.0—making metabolomics more meaningful. Nucleic Acids Res. 43, W251–W257 (2015). 25897128 10.1093/nar/gkv380 PMC4489235

[b78] Colinet H., Larvor V., Bical R. & Renault D. Dietary sugars affect cold tolerance of *Drosophila melanogaster*. Metabolomics 9, 608–622 (2013).

