# Supplementary material for: Cold tolerance is unaffected by oxygen availability despite changes in anaerobic metabolism
Source: Sci Rep. 2016 Sep 13;6:32856. doi: 10.1038/srep32856 (PMC5020647; doi:10.1038/srep32856)
Supplement: Supplementary Information [file srep32856-s1.pdf]

Cold tolerance is unaffected by oxygen availability despite changes in anaerobic metabolism

Leigh Boardman[1†*], Jesper G. Sørensen[2], Vladimír Koštál[3], Petr Šimek[3], and John S. Terblanche[1]

[1] Department of Conservation Ecology and Entomology, Centre for Invasion Biology, Stellenbosch University, Private Bag X1, Matieland, 7602, South Africa

[2] Section for Genetics, Ecology & Evolution, Department of Bioscience, Aarhus University, Ny Munkegade 116, DK-8000 Aarhus C, Denmark

[3] Institute of Entomology, Biology Centre of the Czech Academy of Sciences, České Budějovice, Czech Republic

† Present address: Department of Entomology and Nematology, University of Florida, Gainesville, FL 32611

*Author for correspondence (boardman.leigh@gmail.com)

Additional critical thermal minimum (CT_{min}) results

P_{O2} had no effect on the temperature at which minimum metabolic rate (MetMin) occurred (TMetMin) ($F_{5,56}$ = 0.87, P = 0.51 and $H_{5,66}$ = 7.19, P = 0.21; Table S1), but did affect average metabolic rate (MetAve) and MetMin ($H_{5,65}$ = 29.64, $P < 0.0001$; $H_{5,64}$ = 36.33, $P < 0.0001$; Table S1). Overall, MetAve and MetMin decreased as P_{O2} decreased with the lowest values observed at 0 kPa O_2, but these variables were not significantly different between 10, 21 or 40 kPa O_2. At 2.5 kPa, MetAve and MetMin were significantly higher than at 0 kPa, but not significantly different from 10, 21 or 40 kPa (Table S1).

Additional critical oxygen partial pressure (Pcrit) results

Matched pairs t-tests on both resting and minimum $\dot{V}CO_2$ showed that $\dot{V}CO_2$ was not significantly different at P_{O2} between 5 and 40 kPa O_2. Below 5 kPa, $\dot{V}CO_2$ decreased with a reduction in P_{O2}. $\dot{V}CO_2$ at 2.5 kPa O_2 was significantly lower than at the higher P_{O2}, and at 0 kPa O_2 $\dot{V}CO_2$ was significantly lower than at 2.5 kPa O_2. This suggests a P_{crit} below 5 kPa O_2. According to the linear regression method, P_{crit} was 6 kPa (resting $\dot{V}CO_2$) or 4.5 kPa (minimum $\dot{V}CO_2$) O_2 (see red arrow Fig. 1A). The regression tree analysis yielded P_{crit} values of 3.75 kPa (resting $\dot{V}CO_2$) or 1.25 kPa O_2 (minimum $\dot{V}CO_2$) (see blue arrow Fig. 1A).

Data obtained from P_{crit} experiments was used to measure the degree of spiracle activity by calculating the coefficient of variation of $\dot{V}CO_2$ (COV)[1] from the central 20 min of $\dot{V}CO_2$ from each individual, under each P_{O2}. P_{O2} influenced spiracle behaviour as evidenced by $\dot{V}CO_2$ respirometry traces (Fig. S2) and the COV (Fig. 1B). Under normoxia, as the temperature decreased, metabolic rate and spiracle activity gradually decreased until spiracle activity ceased at $\dot{V}CO_2$ CT_{min} (Fig. S2B). Under 5 kPa O_2, larval burst-interburst gas exchange patterns were short and rapid, with low variability in the respirometry trace indicating small changes in spiracle control (Fig. S2D) and a decrease in COV (Fig. 1B). As P_{O2} decreased further, larval respiration appeared more cyclic in pattern, with longer periods of spiracle opening (Fig. S2E). In some cases, larvae also closed their spiracles for short periods between bursts although gas exchange rarely reached zero. At 0 kPa O_2 no spiracle activity was evident (Fig. S2F).

Excretion events during cooling (thermolimit respirometry)

Excretion events thermolimit respirometry (indicated by a $\dot{V}H_2O$ burst without an increase in $\dot{V}CO_2$) were scored as presence or absence. Cooling under different P_{O2} affected the excretion events in each individual (X^2 = 3.96, d.f. =1, P = 0.046; Fig. S3). Under hypoxia (2.5 and 5 kPa O_2 collectively), only 1 individual out of 18 had an excretion event during the TLR cooling experiment. At 0, 10, 21 and 40 kPa O_2, the number of larvae with and without excretion events did not differ.

Body water and body lipid content after freezing

In order to identify changes in larval body composition after freezing (supercooling point, SCP) under altered oxygen conditions, the body water content (BWC) and body lipid content (BLC) of larvae supercooled under the different P_{O2} were measured using gravimetric means (see Boardman et al., 2013 for detailed methodology). Larvae were baked dry to obtain dry mass before lipids were extracted using chloroform: methanol solution. The difference between fresh mass and dry mass was deemed to be BWC, while the difference between fat-free and dry masses was calculated as BLC. A minimum of n = 16 per P_{O2} were assayed. Differences in BWC and BLC after SCP were analysed using an ANCOVA, as mass was significantly correlated with both BWC and BLC (r = 0.97, P < 0.0001 and r = 0.86, P < 0.0001). The BWC of different groups of larvae after the SCP assay at different P_{O2} was similar ($F_{4,90}$ = 0.20, P = 0.94; Fig. S3b), although BLC differed between the groups ($F_{4,90}$ = 7.05, P < 0.0001; Fig. S1c). After anoxia, BLC was 1 mg (or $\frac{1}{5}$) lower than after the other P_{O2} treatments (Fig. S1c).

Additional metabolomic profiling results

Hyperoxic low temperature effects

Under hyperoxia, the three timepoints separated from one another, but the separation was not significant (permutation test, P = 0.791, Fig. S4A). The metabolites that contributed the most to component 1 (44.7%) were lactic acid, proline, 2-ketoglutaric acid, glutamate, alanine and stearic acid (Fig. S4B). While lactic acid and 2-ketoglutaric acid were not significantly different between timepoints (Table 2, Fig. S7), the concentration of glutamate and stearic acid increased significantly over the course of the experiment (before chill coma < during chill coma < after chill coma, Table 2, Fig. S5, Fig. S8).
Proline was significantly higher during recovery than before or during chill coma (both P<0.0037, Table 2, Fig. S5). The concentration of alanine was significantly higher during chill coma, and after recovery from chill coma (both P<0.0037), than before chill coma (Table 2, Fig. S5).

Anoxic low temperature effects

Under anoxia, the timepoints were not significantly different from one another (permutation test, P = 0.459, Fig. S4K). Recovery was separated from the other two timepoints based on component 1 (32.3%), which loads with margaric acid, glutamine, lactic acid and glutathione (GSH) (Fig. S4L). The concentration of margaric acid was not significantly different between timepoints (Table 2, Fig. S8). Glutamine concentration decreased during chill coma (P<0.0037), before it increased during recovery to levels above before chill coma (P<0.001, Table 2, Fig. S5). The concentration of lactic acid followed the opposite pattern, first increasing significantly during chill coma (P<0.001), and then decreasing significantly below before chill coma concentrations during recovery (P<0.001, Table 2, Fig. S8). Glutathione concentration was not significantly different between timepoints (P>0.0037, Table 2, Fig. S6).

References

1. Lighton, J. R. B. & Lovegrove, B. G. A temperature-induced switch from diffusive to convective ventilation in the honeybee. *J. Exp. Biol.* **154,** 509–516 (1990).

2. Boardman, L., Grout, T. G. & Terblanche, J. S. False codling moth *Thaumatotibia leucotreta* (Lepidoptera, Tortricidae) larvae are chill-susceptible. *Insect Sci.* **19,** 315–328 (2012).

3. Benjamini, Y. & Hochberg, Y. Controlling the False Discovery Rate: A Practical and Powerful Approach to Multiple Testing. *J. R. Stat. Soc. Ser. B* **57,** 289–300 (1995).

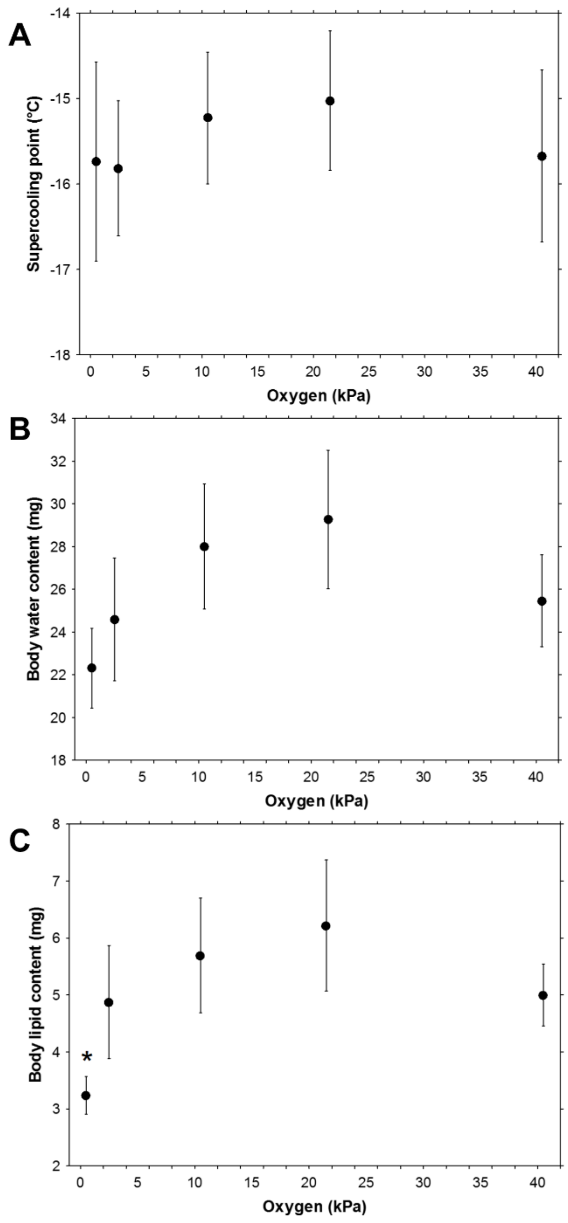

Figure S1. Supercooling point (A) was determined for *T. leucotreta* larvae under five different P_{O2} conditions. Thereafter, body water content (B) and body lipid content (C) were determined. Graph shows mean ± 95 % confidence intervals. * indicates where variable was significantly different from the other P_{O2} conditions.

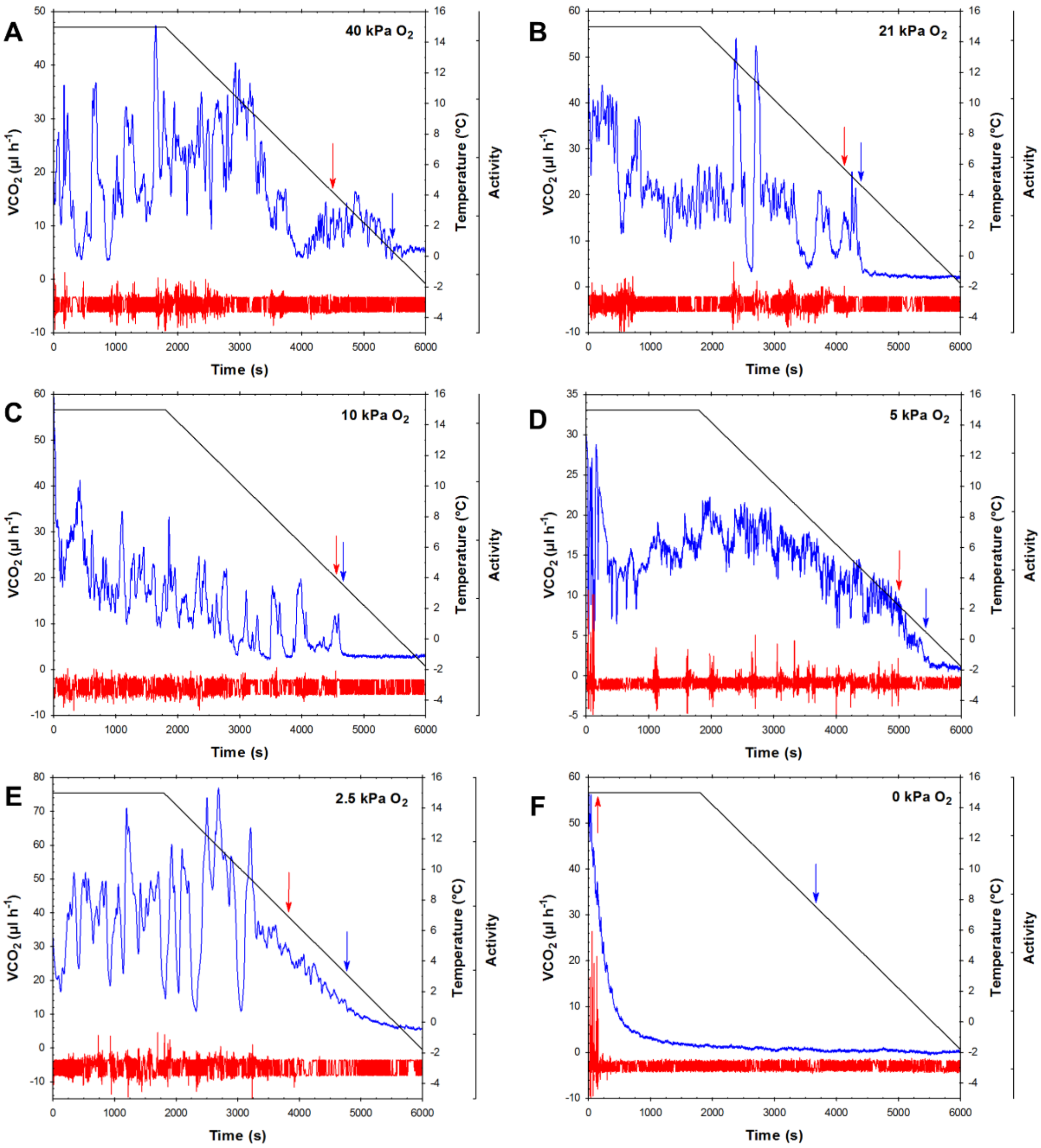

Figure S2. Representative examples of thermolimit respirometry (TLR) traces for critical thermal minima (CT_{min}) of *T. leucotreta* larvae under different P_{O2} conditions: A) 40 kPa O_2, B) 21 kPa O_2, C) 10 kPa O_2, D) 5 kPa O_2, E) 2.5 kPa O_2 and F) 0 kPa O_2. $\dot{V}CO_2$ in µl.h^{-1} is shown in blue (left axis), temperature (°C) is black (right axis), activity (in arbitrary units) is red (right secondary axis). Blue arrows indicate $\dot{V}CO_2$ CT_{min} and red arrows indicate activity CT_{min}. Flow rate was 200ml.ml^{-1}. Individual masses of the represented larvae are: A) 67.4 mg, B) 36.2 mg, C) 34.1 mg, D) 45.2 mg, E) 56.5 mg, F) 51.2 mg. Note that axes are scaled differently for each panel.

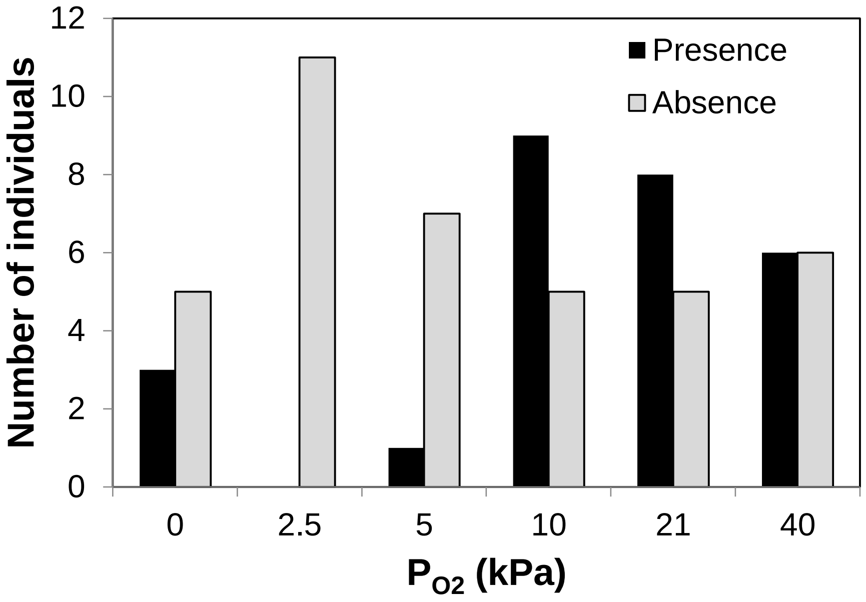

Figure S3. Presence or absence of excretion events recorded during thermolimit respirometry under each of the P_{O2} conditions.

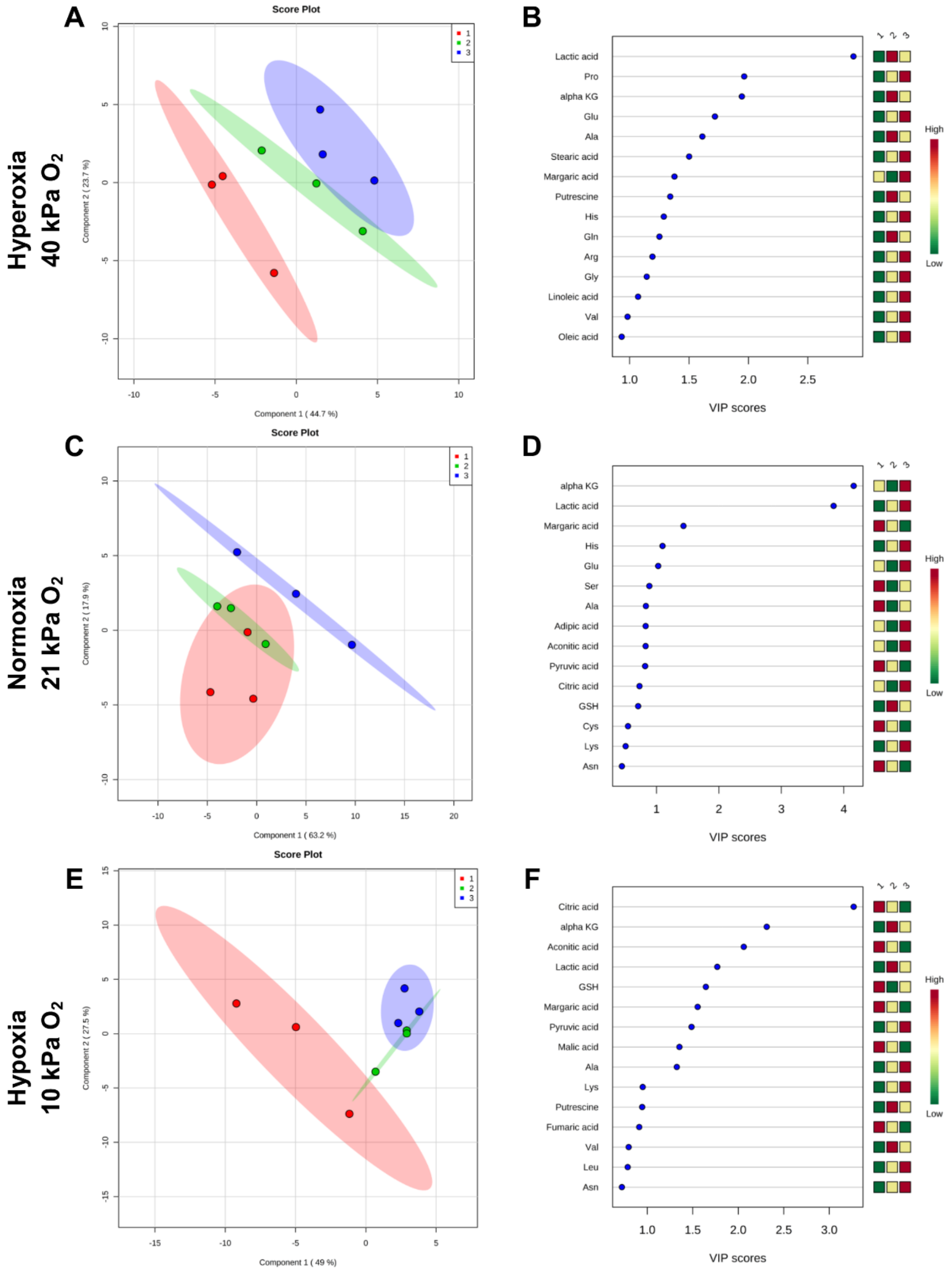

Figure S4. Continued on next page…

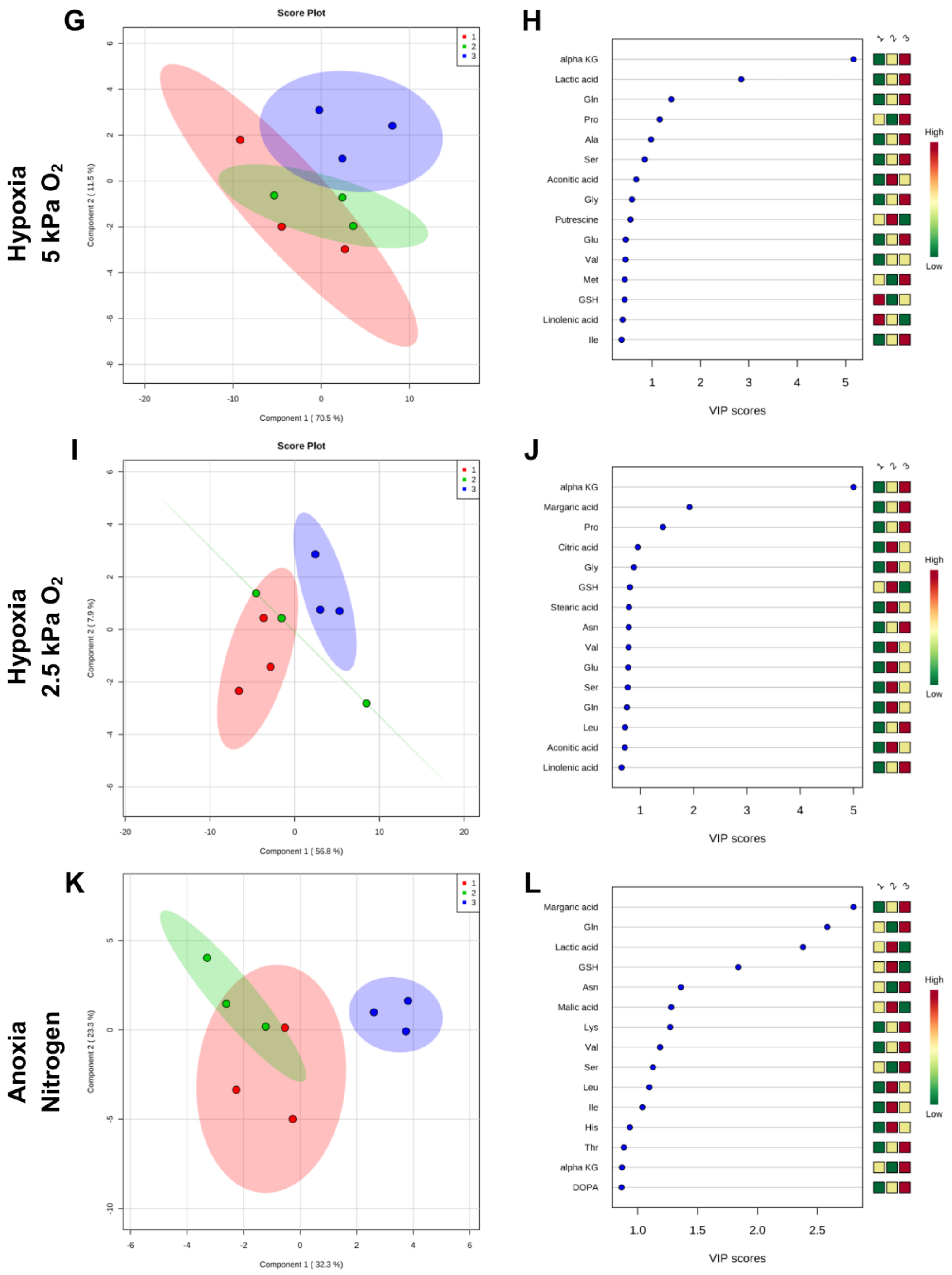

Figure S4. 2D score plots (A, C, E, G, I, K) showing the projection of samples onto the first discriminant plane for each of the six oxygen treatments used (PLS-DA analysis in MetaboAnalyst). Each point is a sample, colours represent different timepoints: red - before chill coma, green – during chill coma, blue – after chill coma and lines indicate 95% confidence intervals. None of the results of the PLS-DA resulted in significant separation (permutation test, $P > 0.05$). The variable importance plots (B, D, F, H, J, L) based on VIP scores shows the top 15 variables that contribute to component 1 at each of the oxygen treatments (based on Pearson correlation). The coloured boxes on the right indicate the relative concentrations of each of the metabolites in each oxygen treatment, with reds indicating highest concentrations and greens the lowest concentrations. Timepoint 1: before chill coma, 2: during chill coma and 3: after chill coma.

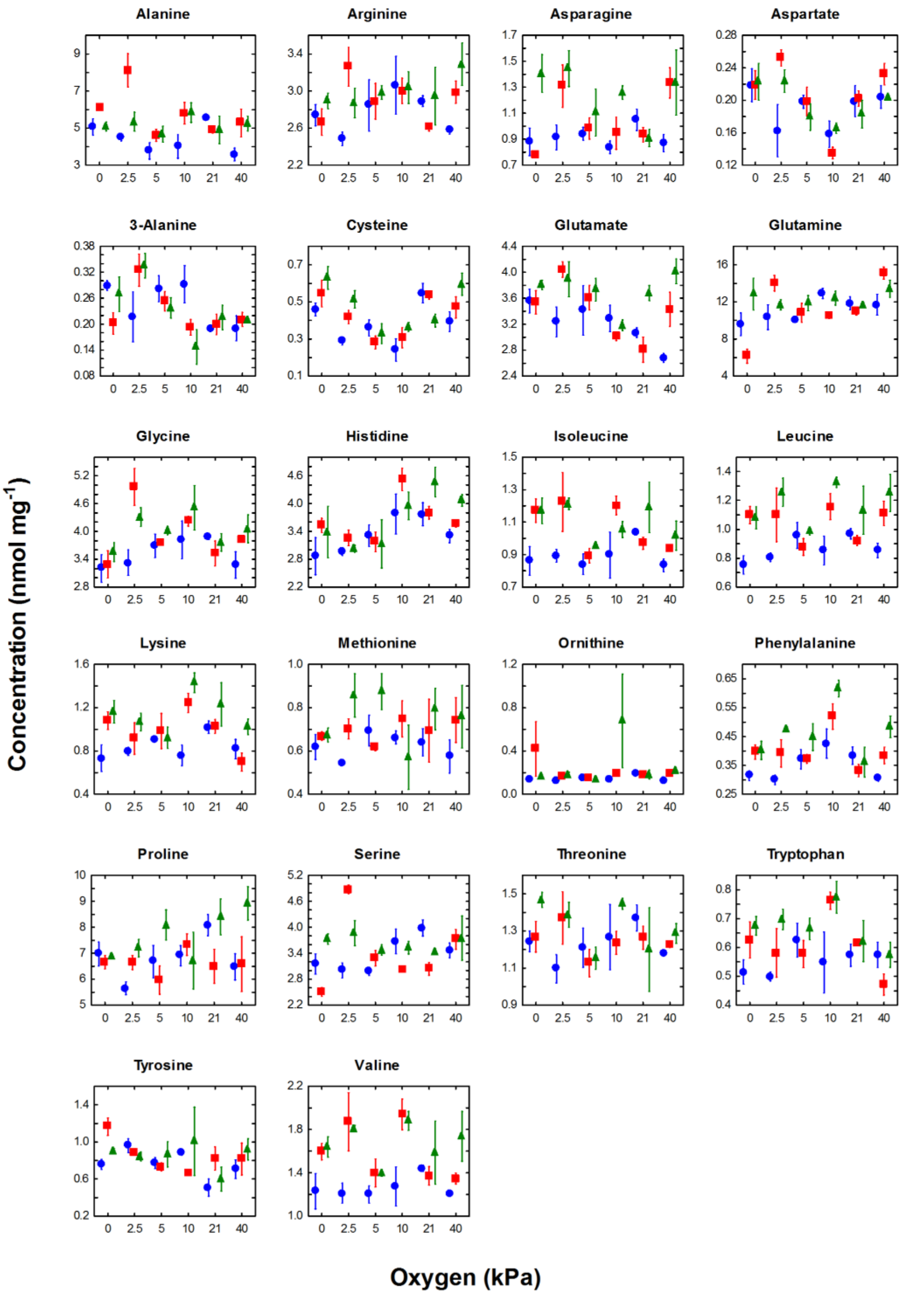

Figure S5. Mean concentrations (± S.E.M.) of the 22 amino acids detected in *Thaumatotibia leucotreta* under each of the oxygen treatments. Before chill coma samples are represented by blue circles, during chill coma by red squares and after chill coma by green triangles. Significant differences are shown in Tables 2 and S3. Metabolites from four individuals were pooled for each sample. Three samples per oxygen and timepoint were analysed.

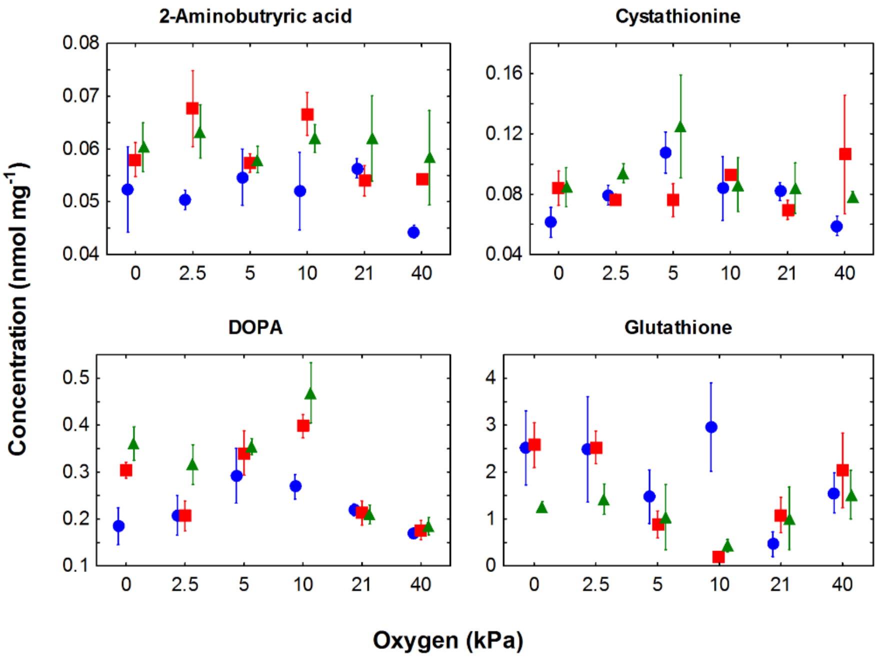

Figure S6. Mean concentrations (± S.E.M.) of 3 additional amino acids and glutathione detected in *Thaumatotibia leucotreta* under each of the oxygen treatments. Before chill coma samples are represented by blue circles, during chill coma by red squares and after chill coma by green triangles. Significant differences are shown in Tables 2 and S3. Metabolites from four individuals were pooled for each sample. Three samples per oxygen and timepoint were analysed.

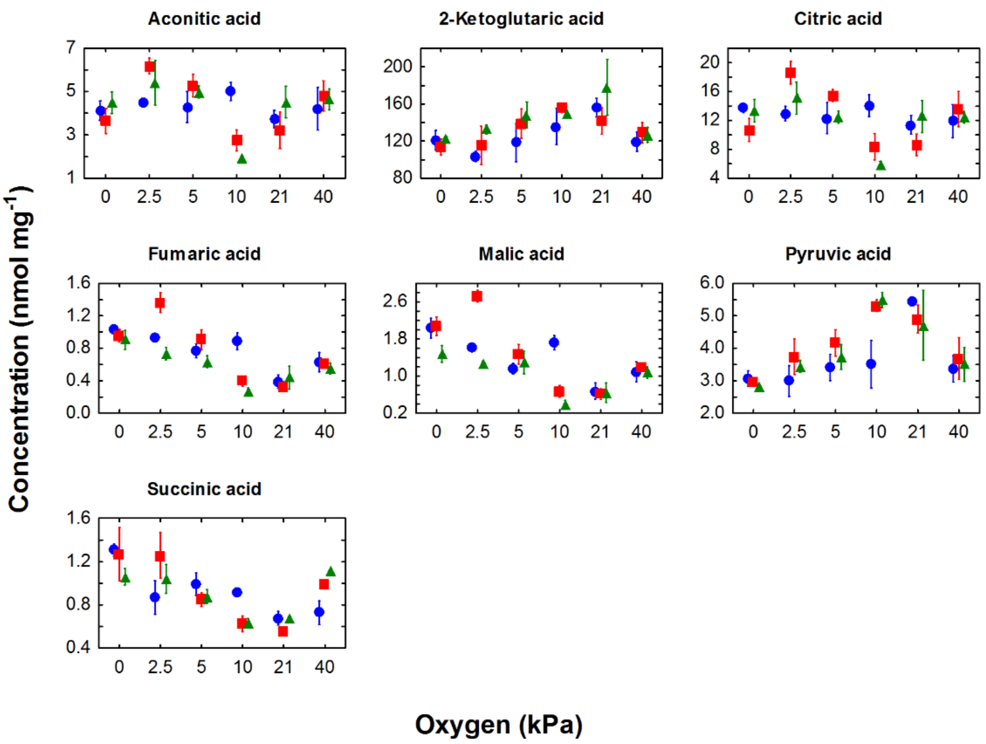

Figure S7. Mean concentrations (± S.E.M.) of the 7 tricarboxylic (TCA) cycle metabolites detected in *Thaumatotibia leucotreta* under each of the oxygen treatments. Before chill coma samples are represented by blue circles, during chill coma by red squares and after chill coma by green triangles. Significant differences are shown in Tables 2 and S3. Metabolites from four individuals were pooled for each sample. Three samples per oxygen and timepoint were analysed.

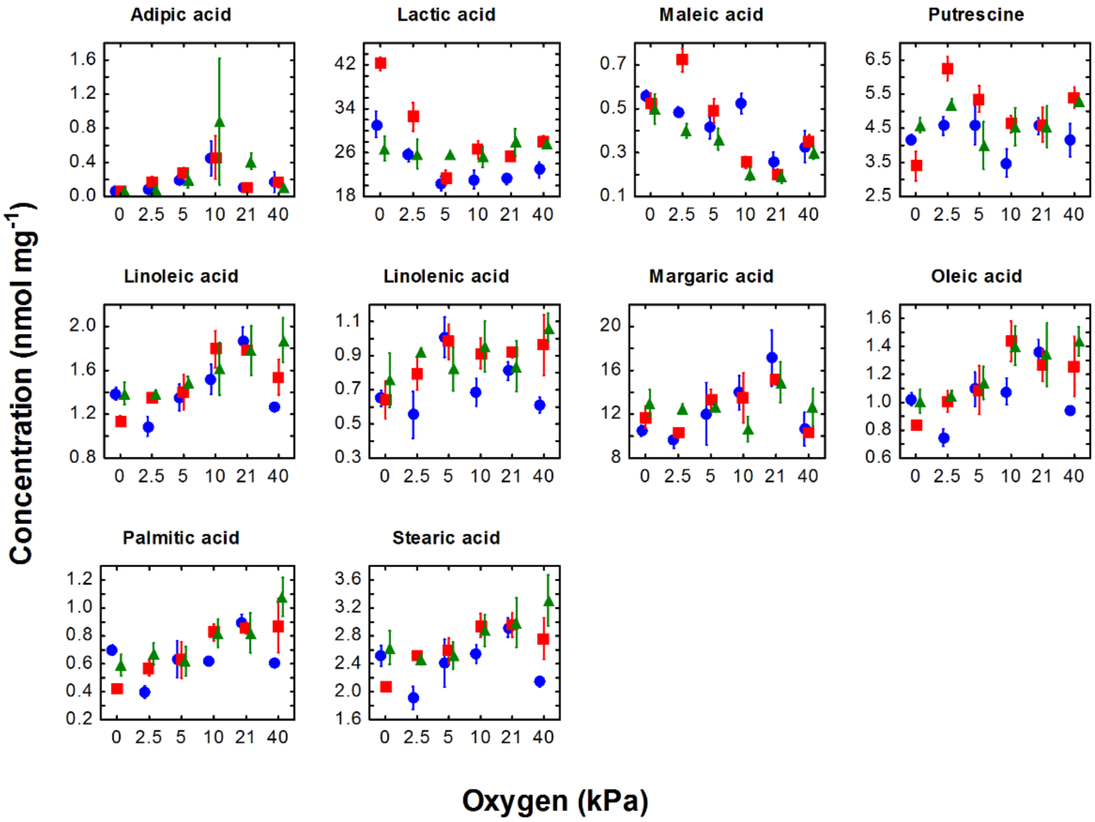

Figure S8. Mean concentrations (± S.E.M.) of the 9 organic acids and putrescine detected in *Thaumatotibia leucotreta* under each of the oxygen treatments. Before chill coma samples are represented by blue circles, during chill coma by red squares and after chill coma by green triangles. Significant differences are shown in Tables 2 and S3. Metabolites from four individuals were pooled for each sample. Three samples per oxygen and timepoint were analysed.

Table S1. Additional results (extended Table 1) of critical thermal minima determined by thermolimit respirometry (TLR) under different P_{O2} conditions. Average and minimum metabolic rates during cooling from 15 °C to 0 °C are also presented. Where statistics indicated a significant effect of P_{O2}, different letters between P_{O2} indicate significant differences based on non-overlapping 95 % confidence intervals. Data indicates mean ± S.E.M.

**O_2 (kPa)**	**Visual CT_{min}* (°C)**	**Mass (mg)**	**$\dot{V}CO_2$ CT_{min} (°C)**	**Activity CT_{min} (°C)**	**MetAve (CO_2, µl h^{-1})**	**MetMin (CO_2, µl h^{-1})**	**TMetMin (°C)**
0		38.69±7.66 (n=8)	7.33±2.08 (n=8)	Undetectable (n=4)	1.19±0.69a (n=8)	-0.62±0.33†a (n=8)	4.06±1.64 (n=8)
2.5		50.25±6.88 (n=11)	4.27±0.78 (n=11)	4.54±1.00 (n=9)	18.23±1.82c (n=11)	6.10±0.49c (n=10)	2.79±1.18 (n=11)
5		43.53±9.37 (n=8)	3.82±1.64 (n=6)	3.93±1.28 (n=8)	5.69±2.12ab (n=8)	0.23±0.50ab (n=8)	3.02±0.86 (n=8)
10		44.84±9.48 (n=14)	5.03±0.49 (n=14)	7.09±1.48 (n=6)	15.44±3.00bc (n=13)	3.73±1.11bc (n=13)	3.52±0.78 (n=14)
21	6.7±0.1 (n=19)	40.31±6.70 (n=13)	5.45±0.53 (n=12)	5.10±0.69 (n=9)	20.92±6.92bc (n=13)	6.66±3.30abc (n=13)	2.06±0.62 (n=13)
40		44.99±10.87 (n=12)	4.65±0.72 (n=12)	5.80±1.20 (n=5)	14.75±3.84bc (n=12)	3.90±1.59bc (n=12)	2.78±0.76 (n=12)
		$F_{4,50}$ = 1.04, *P* = 0.40	$F_{5,56}$ = 0.87, *P* = 0.51	$H_{4,37}$ = 3.48, *P* = 0.48	$H_{5,65}$ = 29.64, ***P* < 0.0001**	$H_{5,64}$ = 36.33, ***P* < 0.0001**	$H_{5,66}$ = 7.19, *P* = 0.21

$\dot{V}CO_2$ CT_{min} and activity CT_{min} were calculated from the inflection point of regressions of the absolute difference sum (ADS) residuals (see methods). Thermolimit data: average metabolic rate during cooling (MetAve), minimum metabolic rate during cooling (MetMin) and temperature at which MetMin occurred (TMetMin).

*Critical thermal minimum (CT_{min}) data from Boardman *et al.*[2]

†Not significantly different from analyser baseline

Table S2. List of metabolites detected in *Thaumatotibia leucotreta* and summary of the results of generalised linear model (GLZ) for the effects of oxygen and timepoint (before-, during-, or after chill coma) on each of the metabolites detected. Significant effects (*P*<0.0156 after false discovery rate correction using the Benjamini-Hochberg procedure[3]**)** are highlighted in bold font.

**KEGG**	**Metabolite**	**Oxygen**			**Timepoint**			**Oxygen*Timepoint**		
		DF	Wald X^2	*P*-value	DF	Wald X^2	*P*-value	DF	Wald X^2	*P*-value
	Amino acids and peptides									
C00041	Alpha-Alanine (Ala)	5	30.73	**<0.0001**	2	36.72	**<0.0001**	10	43.36	**<0.0001**
C00062	Arginine (Arg)	5	6.81	0.24	2	8.34	**0.02**	10	24.3	**<0.01**
C00152	Asparagine (Asn)	5	18.44	**<0.01**	2	36.36	**<0.0001**	10	35.36	**<0.001**
C00049	Aspartate (Asp)	5	52.18	**<0.0001**	2	4.71	0.09	10	28.32	**<0.01**
C00099	Beta-Alanine (3-Ala)	5	34.37	**<0.0001**	2	0.9	0.64	10	41.85	**<0.0001**
C00736	Cysteine (Cys)	5	94.61	**<0.0001**	2	16.27	**<0.001**	10	39.9	**<0.0001**
C00025	Glutamate (Glu)	5	35.97	**<0.0001**	2	33.41	**<0.0001**	10	42.29	**<0.0001**
C00064	Glutamine (Gln)	5	48.98	**<0.0001**	2	10.38	**<0.01**	10	68.71	**<0.0001**
C00037	Glycine (Gly)	5	33.22	**<0.0001**	2	18.38	**<0.001**	10	26.9	**<0.01**
C00135	Histidine (His)	5	51.93	**<0.0001**	2	8	0.02	10	14.7	0.14
C00407	Isoleucine (Ile)	5	24.31	**<0.001**	2	32.61	**<0.0001**	10	18.57	0.05
C00123	Leucine (Leu)	5	11.35	0.04	2	52.5	**<0.0001**	10	22.64	**0.01**
C00047	Lysine (Lys)	5	26.17	**<0.0001**	2	38.43	**<0.0001**	10	27.58	**<0.01**
C00073	Methionine (Met)	5	2.6	0.76	2	10.84	**<0.01**	10	16.69	0.08
C00077	Ornithine (Orn)	5	8.08	0.15	2	4.4	0.11	10	20.09	0.03
C00079	Phenylalanine (Phe)	5	70.43	**<0.0001**	2	54.4	**<0.0001**	10	27.59	**<0.01**
C00148	Proline (Pro)	5	11.31	0.05	2	18.66	**<0.0001**	10	25.84	**<0.01**
C00065	Serine (Ser)	5	42.44	**<0.0001**	2	8.32	**0.02**	10	110.75	**<0.0001**
C00188	Threonine (Thr)	5	9.39	0.09	2	5.1	0.08	10	17.34	0.07
C00078	Tryptophan (Trp)	5	20.74	**<0.001**	2	20.75	**<0.0001**	10	21.97	**0.02**
C00082	Tyrosine (Tyr)	5	15.94	**<0.01**	2	3.1	0.21	10	19.94	0.03
C00183	Valine (Val)	5	20.51	**<0.001**	2	43.4	**<0.0001**	10	20.26	0.03
C02356	2-Aminobutyric acid (2-Aba)	5	7.8	0.17	2	17.38	**<0.001**	10	9.58	0.48
C02291	Cystathionine (CTH)	5	7.88	0.16	2	3.07	0.22	10	14.54	0.15
C00356	3,4-dihydroxyphenylalanine (DOPA)	5	107.46	**<0.0001**	2	32.62	**<0.0001**	10	27.63	**<0.01**
C00051	Glutathione (GSH)	5	20.4	**<0.01**	2	8.69	**0.01**	10	24.24	**<0.01**
	TCA cycle metabolites									
	Aconitic acid	5	37.22	**<0.0001**	2	0.01	1.00	10	36.36	**<0.0001**
C00026	2-Ketoglutaric acid (2-KG)	5	31.54	**<0.0001**	2	6.93	0.03	10	7.61	0.67
C00158	Citric acid	5	39.23	**<0.0001**	2	1.16	0.56	10	41.77	**<0.0001**
C00122	Fumaric acid	5	185.56	**<0.0001**	2	25.25	**<0.0001**	10	67.6	**<0.0001**
C00711	Malic acid	5	220.69	**<0.0001**	2	38.95	**<0.0001**	10	105.82	**<0.0001**
C00022	Pyruvic acid	5	69.01	**<0.0001**	2	5.13	0.08	10	17.98	0.06
C00042	Succinic acid	5	90.73	**<0.0001**	2	0.24	0.89	10	35.6	**<0.0001**
	Other organic acids									
C06104	Adipic acid	5	20.85	**<0.001**	2	1.7	0.43	10	5.93	0.82
C00186	Lactic acid	5	116.81	**<0.0001**	2	50.12	**<0.0001**	10	70.79	**<0.0001**
C01384	Maleic acid	5	193.38	**<0.0001**	2	32.66	**<0.0001**	10	71.66	**<0.0001**
C01595	Linoleic acid	5	56.96	**<0.0001**	2	8.09	0.02	10	21.32	0.02
C06427	Linolenic acid	5	16.09	**<0.01**	2	13.49	**<0.01**	10	19.8	0.03
	Margaric acid	5	33.92	**<0.0001**	2	0.43	0.81	10	15.02	0.13
C00712	Oleic acid	5	45.18	**<0.0001**	2	11.29	**<0.01**	10	18.54	0.05
C00249	Palmitic acid	5	48.71	**<0.0001**	2	8.01	0.02	10	29.15	**<0.01**
C01530	Stearic acid	5	32.32	**<0.0001**	2	15.85	**<0.001**	10	24.52	**0.0063**
	Biogenic amine									
C00134	Putrescine	5	30.25	**<0.0001**	2	13.05	**<0.01**	10	31.43	**<0.001**

Table S3. Summary of significant differences between oxygen treatments (O_2) within each timepoint (Time). Time 1 is before chill coma, time 2 is during chill coma and time 3 is after chill coma. Significance based on least square means from generalized linear models output for each metabolite. Significant effects (P<0.0049 after false discovery rate correction using the Benjamini-Hochberg procedure[3]) are indicated by coloured blocks. The level of significance is indicated by the colour: white is P>0.0049, yellow is 0.0049>P>0.001, and red is P<0.001.

Group 1: O_2	0	0	0	0	0	0	0	0	0	0	0	0	0	0	0	2.5	2.5	2.5	2.5	2.5	2.5	2.5	2.5	2.5	2.5	2.5	2.5	5	5	5	5	5	5	5	5	5	10	10	10	10	10	10	21	21	21
Group 1: Time	1	1	1	1	1	2	2	2	2	2	3	3	3	3	3	1	1	1	1	2	2	2	2	3	3	3	3	1	1	1	2	2	2	3	3	3	1	1	2	2	3	3	1	2	3
vs																																													
Group 2: O_2	2.5	5	10	21	40	2.5	5	10	21	40	2.5	5	10	21	40	5	10	21	40	5	10	21	40	5	10	21	40	10	21	40	10	21	40	10	21	40	21	40	21	40	21	40	40	40	40
Group 2: Time	1	1	1	1	1	2	2	2	2	2	3	3	3	3	3	1	1	1	1	2	2	2	2	3	3	3	3	1	1	1	2	2	2	3	3	3	1	1	2	2	3	3	1	2	3
Amino acids and peptides																																													
Ala							yellow													red											red	red	red				yellow						yellow		
Arg							yellow									yellow																yellow													
Asn							red			red				yellow									yellow												red									yellow	yellow
Asp	yellow	yellow				red					yellow									red	yellow	yellow	red	yellow							yellow														
3-Ala				yellow	yellow		yellow				yellow							yellow	yellow	yellow				red								yellow	yellow	yellow	yellow	yellow									
Cys	red	yellow				red		red			red		red	red				red				red	yellow				red		red					yellow			yellow		red	yellow		red			yellow
Glu					red				yellow		yellow									red				yellow			yellow					red						yellow	yellow					yellow	
Gln	yellow					red	red	red	red	red										yellow			red								yellow	yellow								red				red	
Gly						yellow	red				yellow																				red	red	yellow												
His						yellow								yellow						yellow	red		yellow												red	yellow					red	yellow			
Ile								yellow													yellow										yellow		yellow												
Leu																									yellow																				
Lys										yellow													red	yellow	red		yellow																		
Met																								yellow	yellow																				
Orn											yellow													yellow	red	yellow	yellow																		
Phe	yellow					yellow					red					yellow			yellow	yellow	yellow	red	yellow	yellow	red	red	yellow								yellow										yellow
Pro															yellow												yellow		yellow																
Ser				yellow			red	yellow		red							yellow			red			yellow						red		red	red	red				red							yellow	
Thr													yellow																																
Trp																				yellow	yellow		red				yellow																		
Tyr						yellow		yellow																		yellow			yellow																
Val																					yellow	yellow	yellow		yellow						yellow	yellow	yellow												

continued on the next page…

Group 1:	O_2	0	0	0	0	0	0	0	0	0	0	0	0	0	0	0	2.5	2.5	2.5	2.5	2.5	2.5	2.5	2.5	2.5	2.5	2.5	2.5	5	5	5	5	5	5	5	5	5	10	10	10	10	10	10	21	21	21
	Time	1	1	1	1	1	2	2	2	2	2	3	3	3	3	3	1	1	1	1	2	2	2	2	3	3	3	3	1	1	1	2	2	2	3	3	3	1	1	2	2	3	3	1	2	3
vs																																														
Group 2:	O_2	2.5	5	10	21	40	2.5	5	10	21	40	2.5	5	10	21	40	5	10	21	40	5	10	21	40	5	10	21	40	10	21	40	10	21	40	10	21	40	21	40	21	40	21	40	40	40	40
	Time	1	1	1	1	1	2	2	2	2	2	3	3	3	3	3	1	1	1	1	2	2	2	2	3	3	3	3	1	1	1	2	2	2	3	3	3	1	1	2	2	3	3	1	2	3
2-Aminobutyric acid (2-Aba)																																														
Cystathionine (CTH)																																														
DOPA																																														
Glutathione (GSH)																																														
TCA cycle metabolites																																														
Aconitic acid																																														
2-Ketoglutaric acid (2-KG)																																														
Citric acid																																														
Fumaric acid																																														
Malic acid																																														
Pyruvic acid																																														
Succinic acid																																														
Other organic acids																																														
Adipic acid																																														
Lactic acid																																														
Maleic acid																																														
Linoleic acid																																														
Linolenic acid																																														
Margaric acid																																														
Oleic acid																																														
Palmitic acid																																														
Stearic acid																																														
Biogenic amine																																														
Putrescine																																														
